# Supplementary material for: Unusual Sabatier principle on high entropy alloy catalysts for hydrogen evolution reactions
Source: Nat Commun. 2024 Jan 8;15:359. doi: 10.1038/s41467-023-44261-4 (PMC10774414; doi:10.1038/s41467-023-44261-4)
Supplement: Supplementary file 1 — Supplementary Information [file 41467_2023_44261_MOESM1_ESM.pdf]

Supplementary Materials for

Unusual Sabatier principle on high entropy alloy catalysts for hydrogen evolution reactions

Zhi Wen Chen[1,2], Jian Li[1], Pengfei Ou[3], Jianan Erick Huang[3], Zi Wen[1], Li Xin Chen[2], Xue Yao[2], Guang Ming Cai[4], Chun Cheng Yang[1*], Chandra Veer Singh[2,5*], Qing Jiang[1*]

Correspondence to: ccyang@jlu.edu.cn, chandraveer.singh@utoronto.ca, and jiangq@jlu.edu.cn

This PDF file includes:

Supplementary Methods
Supplementary Figs. 1-63
Supplementary Notes 1-10
Supplementary Tables 1-4
Supplementary References 1-28

Supplementary Methods

Materials

Iron(III) chloride ($FeCl_3$), Chromium(II) chloride ($CrCl_2$), Cobalt(II) chloride hexahydrate ($CoCl_2·6H_2O$), Nickel(II) chloride hexahydrate ($NiCl_2·6H_2O$), Copper(II) chloride dihydrate ($CuCl_2·2H_2O$), Manganese(II) chloride tetrahydrate ($MnCl_2·4H_2O$) and Chloroplatinic acid ($H_2PtCl_6·6H_2O$) were purchased from Sigma Aldrich. All chemicals are of analytical purity and used without further purification.

Synthesis of PtFeCoNiCu HEA catalyst

0.5 mmol of $FeCl_3$, $CoCl_2·6H_2O$, $NiCl_2·6H_2O$, $CuCl_2·2H_2O$ and $H_2PtCl_6·6H_2O$ were added into 40 mL ultrapure water to form a uniform mixture. The mixture was stirred constantly in an oil bath at 80 °C until all water evaporated, forming a dark yellow slurry. The HEA-300, HEA-400 HEA-500, HEA-700 and HEA-900 were obtained through annealing the slurry under 5% H_2/Ar atmosphere for 2 h at 300, 400, 500, 700 and 900 °C, respectively.

Synthesis of PtFeCoNi and PtFeCo catalysts

The synthesis method of PtFeCoNi is the same with that of HEA-400, except that the precursor of $CuCl_2·2H_2O$ was not added. The synthesis method of PtFeCo is the same with that of HEA-400, except that the precursors of $CuCl_2·2H_2O$ and $NiCl_2·6H_2O$ were not added.

Synthesis of FeCoNiCrMn HEA catalyst

0.5 mmol of $FeCl_3$, $CoCl_2·6H_2O$, $NiCl_2·6H_2O$, $CrCl_2$ and $MnCl_2·4H_2O$ were added into 40 mL ultrapure water to form a uniform mixture. The mixture was stirred constantly in an oil bath at 80 °C until all water evaporated, forming a dark gray slurry. The FeCoNiCrMn HEA was obtained through annealing the slurry under 5% H_2/Ar atmosphere for 2 h at 500 °C.

Synthesis of FeCoNiCuMn HEA catalyst

0.5 mmol of $FeCl_3$, $CoCl_2·6H_2O$, $NiCl_2·6H_2O$, $CuCl_2·2H_2O$ and $MnCl_2·4H_2O$ were added into 40 mL ultrapure water to form a uniform mixture. The mixture was stirred constantly in an oil bath at 80 °C until all water evaporated, forming a dark yellow slurry. The FeCoNiCuMn HEA was obtained through annealing the slurry under 5% H_2/Ar atmosphere for 2 h at 500 °C.

Synthesis of $PtNi_3$ catalyst

The synthesis of $PtNi_3$ was using the method according to the report of Wang *et al.*(*1*) Typically, 0.4 mmol of $H_2PtCl_6·6H_2O$, 1.2 mmol $Ni(NO_3)_2·6H_2O$ and 287.2 mg polyvinyl pyrrolidone (PVP) were dissolved into 50 mL ultrapure water and sonicated for 1 h. Then, the solution was sprayed onto a glass plate maintained at 400 °C for rapid evaporation. The collected powder was then cleaned through centrifugation for 3 times with ultrapure water. The $PtNi_3$ were obtained through annealing the powder under 5% H_2/Ar atmosphere for 2 h at 500 °C.

Material Characterization

For the structural characterization, X-ray diffraction (XRD) was performed on a D/max2500pc diffractometer with a Cu Kα radiation. X-ray photoelectron spectroscopy (XPS) detection was through an ESCALAB 250Xi spectrometer with a monochromatic Al-K source. The morphology characterization was conducted using a JEM-2100F transmission electron microscope (TEM) for TEM images, high-resolution TEM (HRTEM) images and selected area electron

diffraction (SAED) patterns. The high angle annular dark field (HAADF) images were obtained through a double-corrected FEI Titan Themis 300 electron microscope. The component analysis was confirmed using an inductively coupled plasma optical emission spectroscopy (ICP-OES).

Electrochemical Measurements

All electrochemical measurements were performed on an Ivium-n-Stat electrochemical workstation under a standard three-electrode system. A graphite electrode, a saturated calomel electrode (SCE) and a rotating disk electrode (RDE) covered with catalyst films were used as the counter electrode, reference electrode, and working electrode, respectively. 3 mg of each catalyst powders distributed into 0.5 mL water-isopropanol solution (4:1, v/v) were used as the catalyst ink. 30 μL of the catalyst ink was taken and dried on the RDE surface for measurement each time. 15 μL Nafion-isopropanol solution (1:19, v/v) was taken and covered on the dried catalyst films as a binder.

The HER performance measurements were conducted in N_2-saturated 0.5 M H_2SO_4. For the activation of PtFeCoNiCu, PtFeCoNi, PtFeCo, FeCoNiCrMn and FeCoNiCuMn catalysts, cyclic voltammetry (CV) tests were performed at a potential range of 100 ~ 530 mV (*vs.* reversible hydrogen electrode, RHE) at a scan rate of 400 mV s^{-1}. Linear sweep voltammetry (LSV) tests were conducted at a scan rate of 5 mV s^{-1} with a rotation rate of 2025 rpm. For measuring the double-layer capacitance, CV tests were performed at a potential range of 100 to 200 mV (*vs.* RHE) at scan rates of 20, 40, 60, 80 and 100 mV s^{-1}, respectively. Galvanostatic tests were performed at an applied current density of -10 mA cm^{-2} for 80 h. Electrochemical impedance measurements were performed at 10 mV (*vs.* RHE) from 100 kHz to 0.1 Hz. The hydrogen production was measured with a gas chromatograph (GC-2014), using a thermal conductivity detector (TCD) to detect H_2 content every 10 min. For detecting the amount of produced H_2, 1 mg catalyst was loaded on the Ni foam with sufficient Nafion as the binder. All the measurements were performed at room temperature. All the potentials converted to the RHE were through $E_{RHE} = E_{SCE} + 0.059\ pH + 0.267$ V, where E_{RHE} and E_{SCE} denote the reversible hydrogen evolution potential and the measured potential, respectively.

Turnover frequency calculation

The approach proposed by Jaramillo *et al.* was adopted for the calculation of turnover frequency (TOF) (*2*). The TOF is calculated according to:

$$\text{TOF per site} = \frac{\# \times J \times A}{N \times A} \quad (1)$$

where #, *J*, *A*, and *N* represent hydrogen turn over event, the current density at an applied overpotential, geometric area of the catalyst, and active site number per unit area, respectively. In this work, # is 3.12×10^{15} s^{-1} cm^{-2} per mA cm^{-2} according to the previous report, and *A* is 0.19625 cm^2 corresponding to the geometric area of RDE. It is noted that the surface site density of all materials is reasonably approximated to be 10^{15} cm^{-2}. Therefore, *N* can be calculated through

$$N = C_{dl}/C_s \times 10^{15}\ cm^{-2} \quad (2)$$

where C_{dl} and C_s represent double layer capacitance and specific capacitance. When taking general Cs of 0.035 mF cm^{-2} (*3*), the *N* values for HEA-400, HEA-500, and Pt/C are calculated to be 3.19 × 10^{18}, 1.15 × 10^{18}, and 2.30 × 10^{18} cm^{-2}, respectively. At an overpotential of 20 mV (*vs*. RHE), the *J* values for HEA-400, HEA-500, and Pt/C are 44.6, 22.2, and 7.2 mA cm^{-2}. Thus, the TOF per site at 20 mV (*vs*. RHE) are 4.36 × 10^{-2}, 6.02 × 10^{-2} and 0.98 × 10^{-2} s^{-1} for HEA-400, HEA-500 and Pt/C, respectively.

Density functional theory calculation

All calculations were performed using the Vienna ab initio simulation package (VASP) based on spin-polarized density function theory (DFT) (*4*). The projector-augmented wave pseudopotential was applied to treat the core electrons (*5*). The generalized gradient approximation (GGA) with the Perdew-Burke-Ernzerhof functional (PBE) was adopted in the DFT calculations (*6*). The kinetic energy cutoff for the wave-function calculations was set to 550 eV. The Fermi smearing function was applied with a smearing width of 0.1 eV. The Monkhorst-Pack grid of k-points were $2 \times 2 \times 1$, and a vacuum gap of ~ 15 Å was used to avoid interactions between the system and its mirror images. The geometric relaxation was stopped when the incremental changes in total energy and forces were smaller than 1×10^{-5} eV and 0.05 eV/Å, respectively. The van der Waals interaction was considered through DFT-D3 method proposed by Grimme (*7*). All the transition states were obtained using the climbing image nudged elastic band (CI-NEB) method with the convergence force smaller than 0.05 eV/Å (*8*). To check the accuracy of calculation parameters, higher accuracy parameters were considered to calculate the reaction process of HER on the designed HEA (111), as shown in Supplementary Fig. 59. The relatively small differences (Supplementary Table 4) indicate the reliability of our DFT calculations.

The HEA (111) structures used for DFT calculations were initially created from FCC lattice structures. 4×4 supercell with five layers was selected as the model. The order of the lattice points in each simulation was randomly shuffled and the shuffled lattice points were assigned different types of metal atoms consistent with the corresponding element ratio. The lattice constant of the generated HEA (111) was received by the optimization of geometric structures, and the surface interaction with adsorbates were calculated by relaxing the adsorbates and the top three layers while fixing the bottom two layers. To simulate the strain effect for the adsorption of H*, the strains were obtained by changing the lattice constant of HEA (111) without considering the composition changes.

The adsorption energy (ΔE_{X^*}) of adsorbates (X = H, C, O, N) was calculated by the following equation:

$$\Delta E_{X^*} = E_{X^*} - E_{cat} - E_X \tag{3}$$

where E_{X^*}, E_{cat}, and E_X represent the total energy of the catalyst with the adsorbate, isolated catalyst, and the corresponding adsorbate, respectively. Note that the energies of H, C, O, and N refer to the energies of H_2, graphene, H_2O, and NH_3, respectively. The adsorption energy was corrected by considering the zero-point energy and entropy, as shown below:

$$\Delta G_{X^*} = \Delta E_{X^*} + \Delta ZPE - T\Delta S \tag{4}$$

where ΔG_{X^*}, ΔZPE, and $T\Delta S$ denote the adsorption free energy, zero-point energy change, and entropy change, respectively.

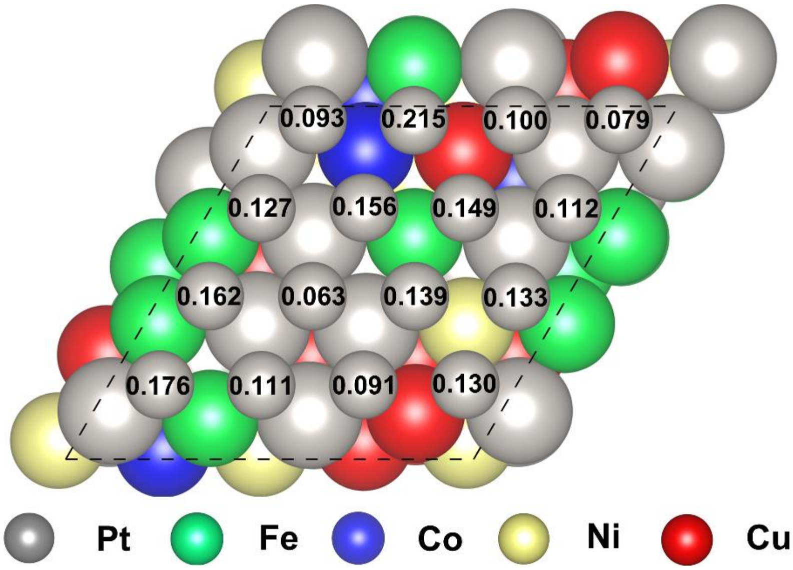

Supplementary Fig. 1. The geometrically optimized structure of PtFeCoNiCu (111). The smaller balls indicate the surface Pt atoms, and the number represents the transferred electron amounts from neighbors to the corresponding Pt atoms.

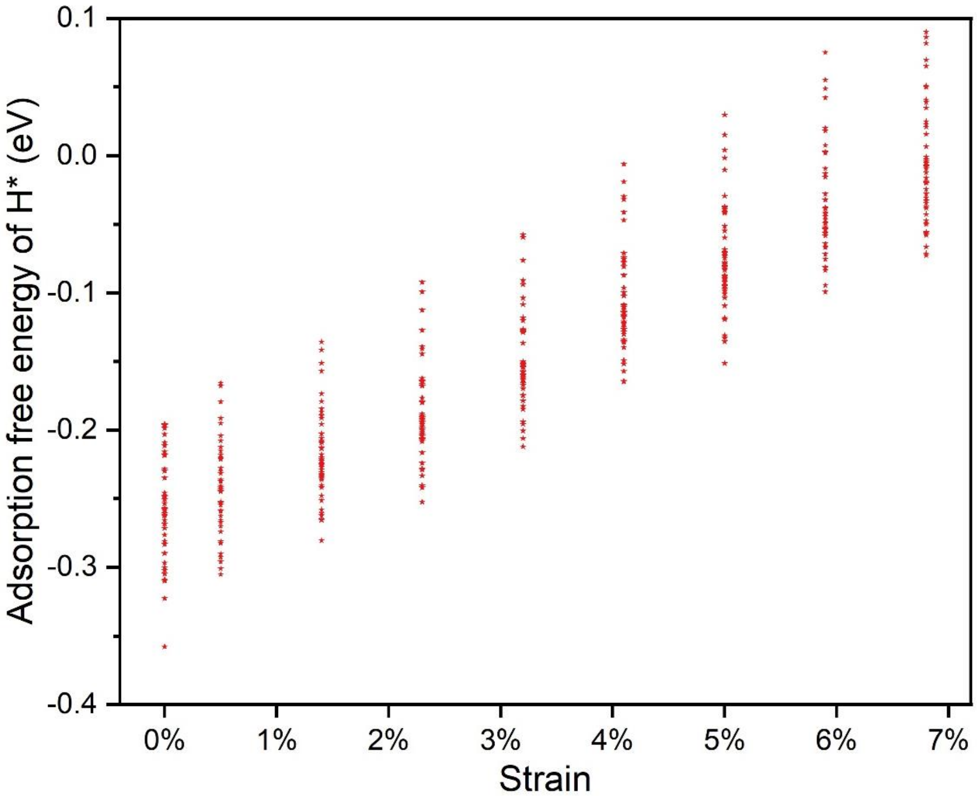

Supplementary Fig. 2. Adsorption free energy of H* (ΔG_{H*}) on random active sites of PtFeCoNiCu HEA systems with different strains.

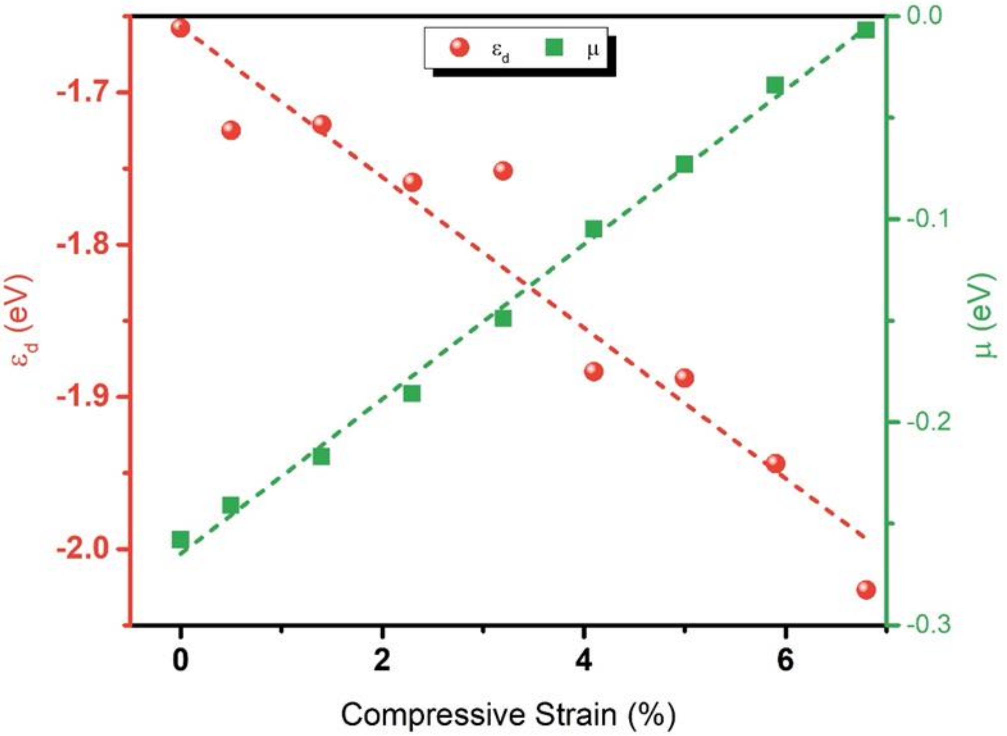

Supplementary Fig. 3. The structure (compressive strain)-property (ε_d, *d*-band center)-performance (μ, the expectation of Gaussian distribution of ΔG_{H*}) relation on HEA catalysts.

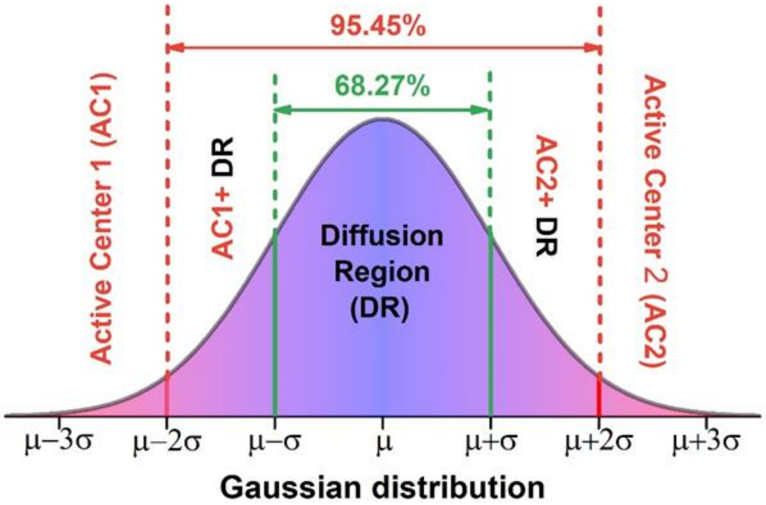

Supplementary Fig. 4. Schematic diagram of Gaussian distribution of ΔG_{H*} on HEA surfaces. Active center 1 (AC1) and active center 2 (AC2) are used for Volmer reaction ($* + H^+ + e^- \rightarrow H*$) and Tafel ($H* + H* \rightarrow H_2$) or Heyrovsky reaction ($H* + H^+ + e^- \rightarrow H_2$), respectively, due to the strong adsorption of H* on AC1 and the weak adsorption of H* on AC2. The region around μ (68.27%) is diffusion region (DR) for H* spillover. There are two coexistence regions of active center and DR between 68.27% and 95.45% in the Gaussian distribution of ΔG_{H*}.

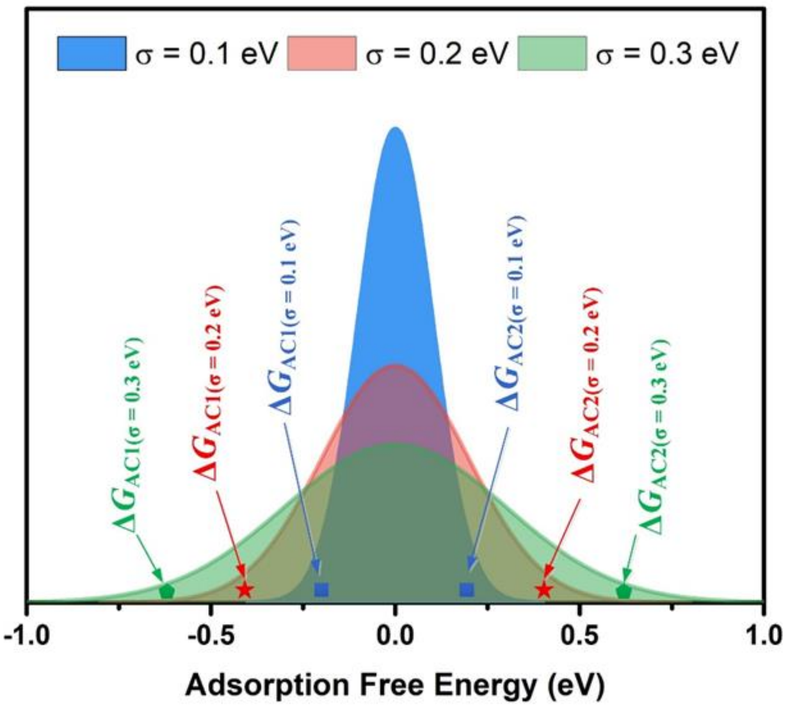

Supplementary Fig. 5. Schematic diagram of Gaussian distribution of adsorption free energy with $\mu = 0$ eV and different σ values (0.1 eV, 0.2 eV, and 0.3 eV).

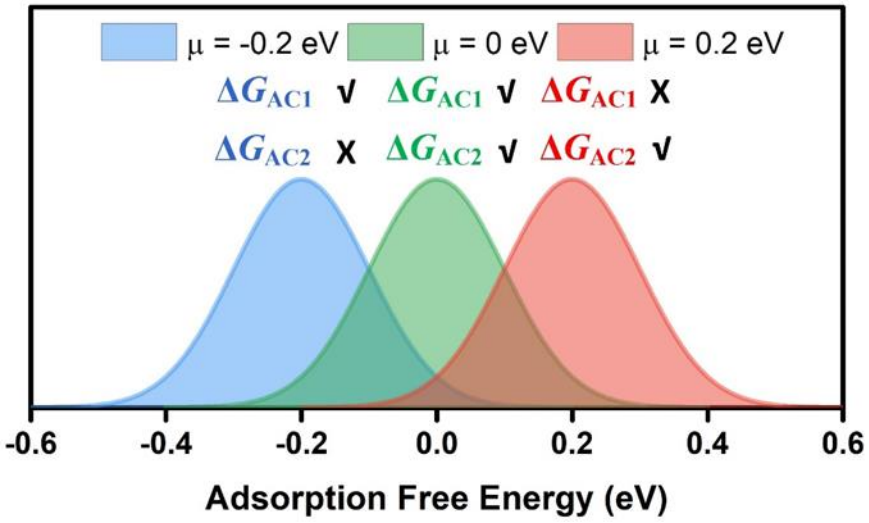

Supplementary Fig. 6. Schematic diagram of Gaussian distribution of adsorption free energy with σ = 0.1 eV and different μ values (-0.2 eV, 0 eV, and 0.2 eV). The potential limiting steps of the three Gaussian distributions are ΔG_{AC2} (μ = -0.2 eV), $\Delta G_{AC1} = \Delta G_{AC2}$ (μ = 0 eV), ΔG_{AC1} (μ = 0.2 eV) for HER. Obviously, the Gaussian distribution with μ = 0 eV has a better catalytic performance than those of the other two.

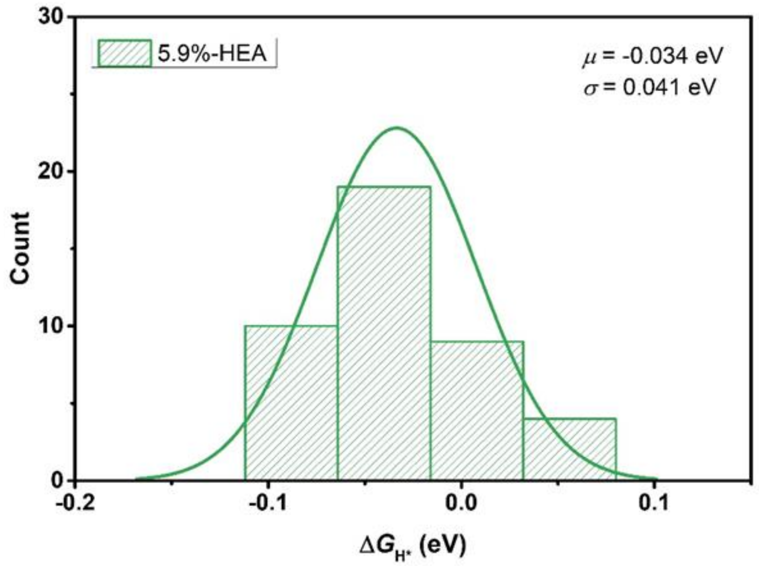

Supplementary Fig. 7. Gaussian distribution of adsorption free energy of H* (ΔG_{H^*}) on 5.9%-HEA.

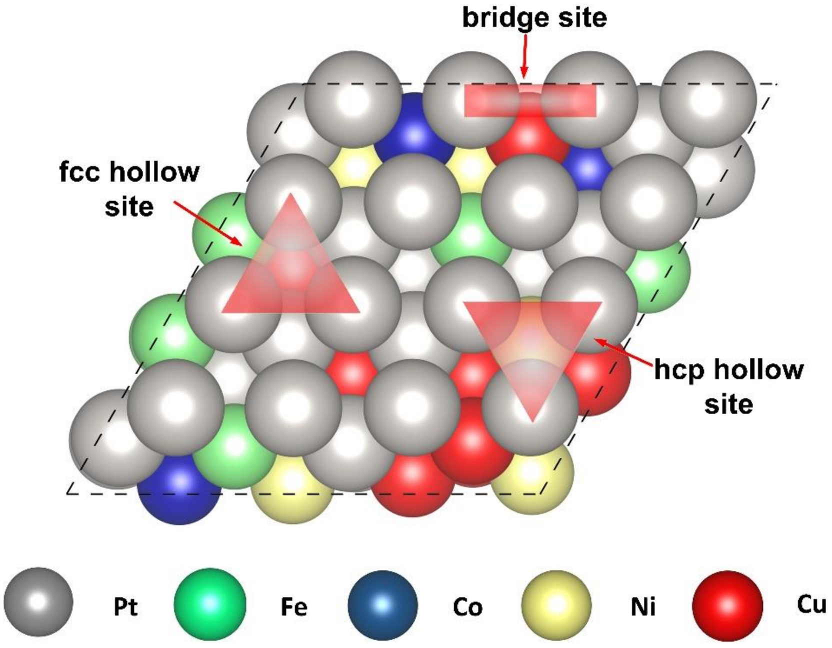

Supplementary Fig. 8. Adsorption site types of the bridge, fcc hollow, and hcp hollow sites for H* on HEA (111).

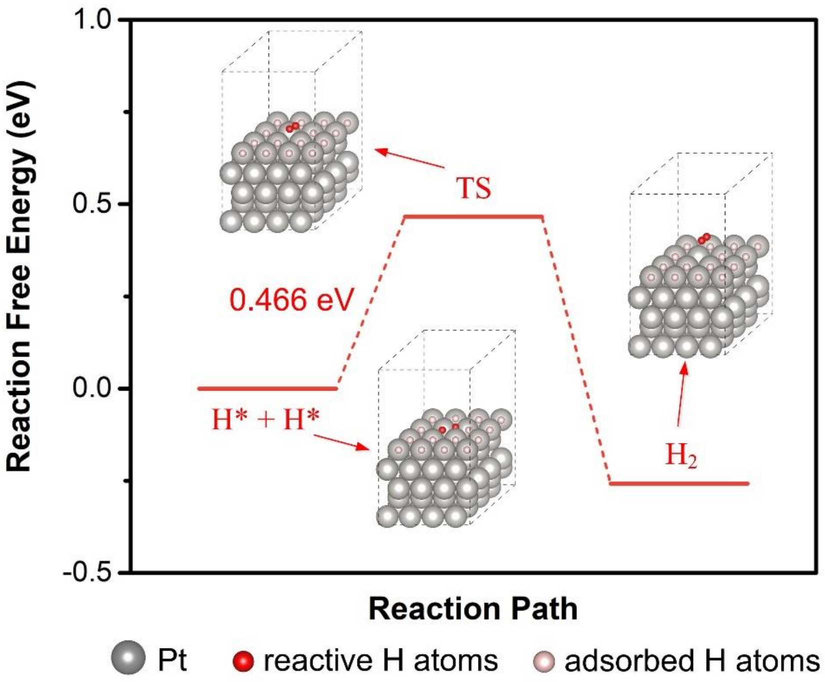

Supplementary Fig. 9. The energy barrier of Tafel reaction ($H^* + H^* \rightarrow H_2$) on Pt (111) with high H* coverage.

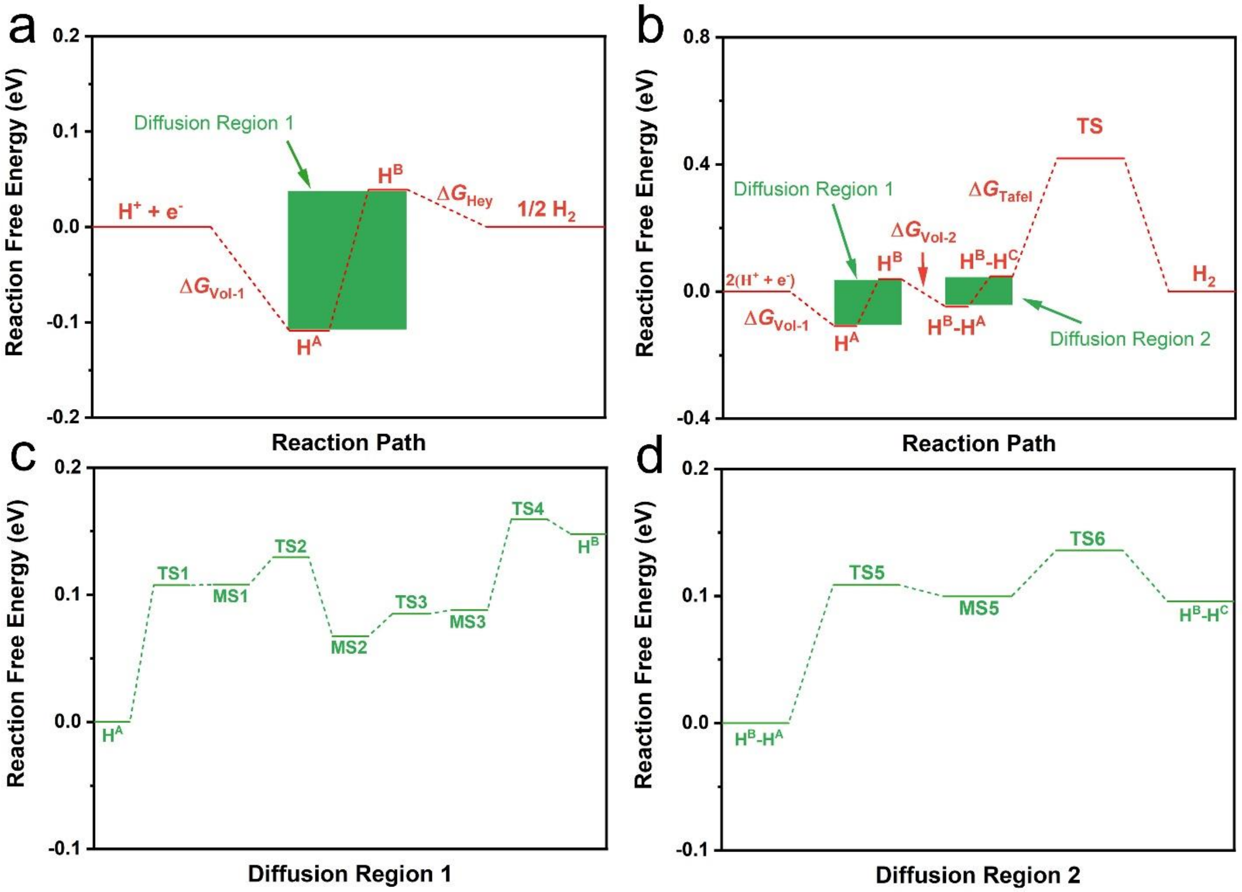

Supplementary Fig. 10. Reaction process of HER on another random active center of 5.9%-HEA (111). (a) Volmer-Heyrovsky mechanism of HER on 5.9%-HEA (111). (b) Volmer-Tafel mechanism of HER on 5.9%-HEA (111). (c) The H* spillover on DR1 (diffusion region for the first H*) for 5.9%-HEA (111). (d) The H* spillover on DR2 (diffusion region for the second H*) for 5.9%-HEA (111).

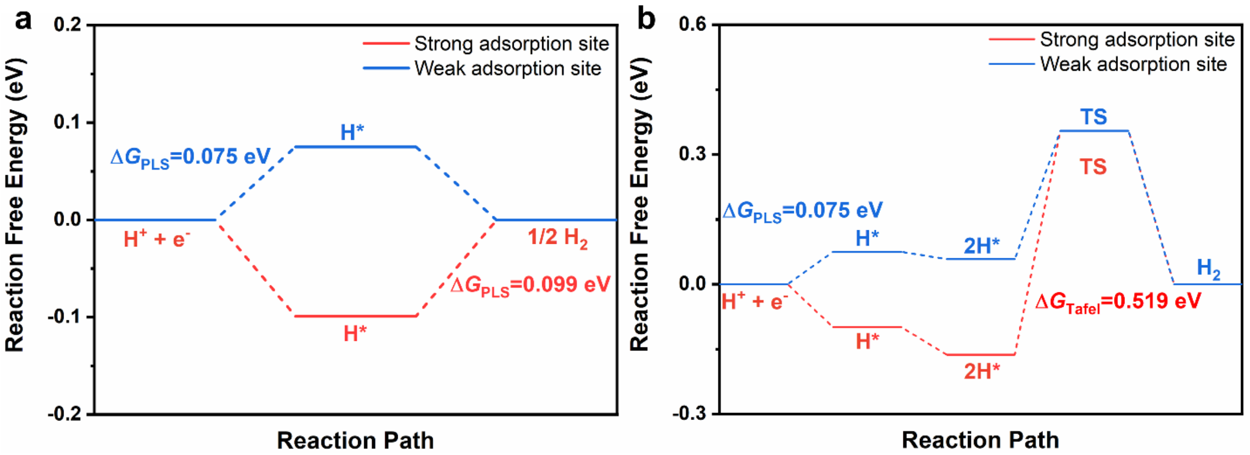

Supplementary Fig. 11. (a) Volmer-Heyrovsky mechanism of HER on 5.9%-HEA (111) without H* spillover. (b) Volmer-Tafel mechanism of HER on 5.9%-HEA (111) without H* spillover.

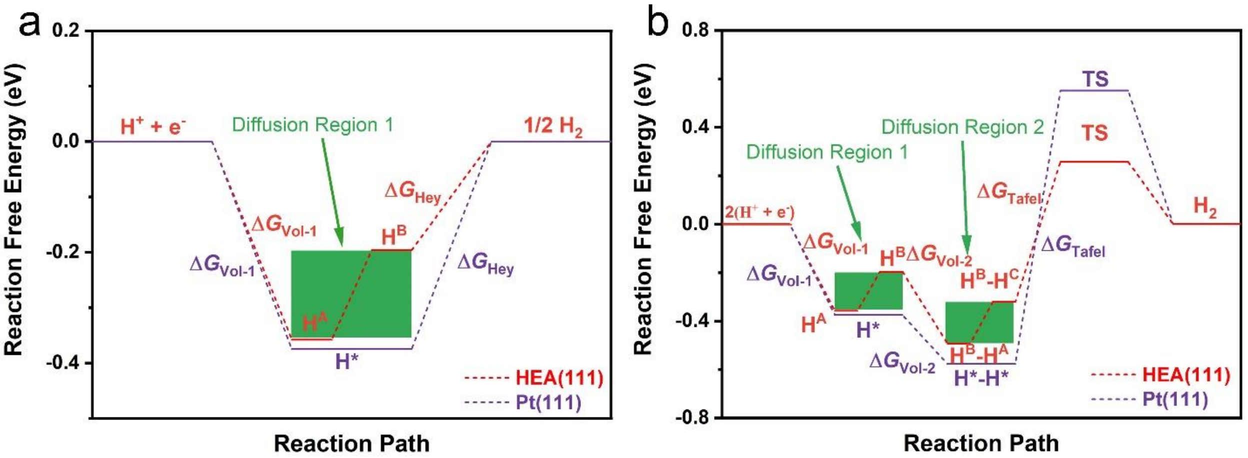

Supplementary Fig. 12. Reaction process of HER on HEA (111) and Pt (111). (a) Volmer-Heyrovsky mechanism of HER on HEA (111) and Pt (111). (b) Volmer-Tafel mechanism of HER on HEA (111) and Pt (111).

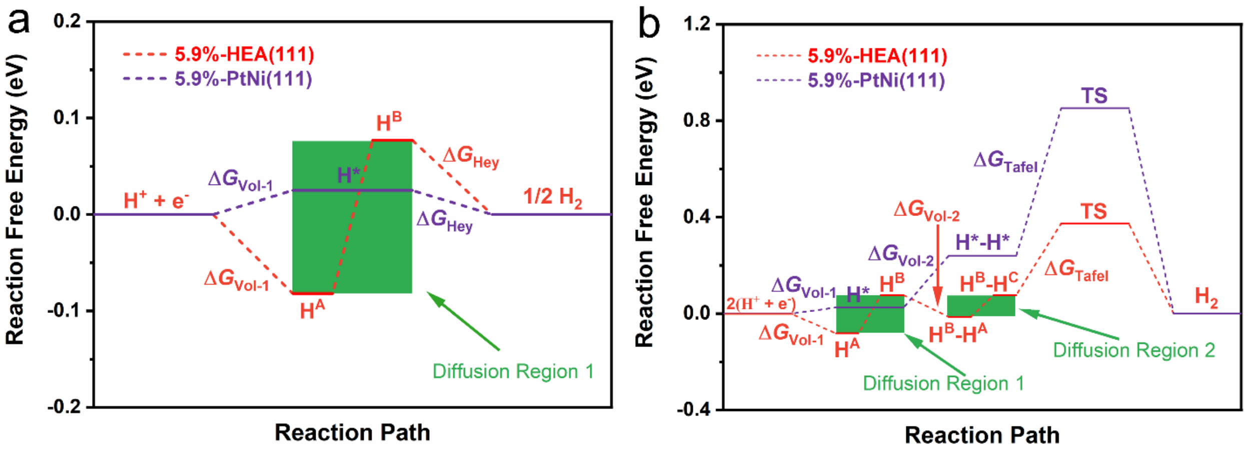

Supplementary Fig. 13. Reaction process of HER on 5.9%-HEA (111) and 5.9%-PtNi (111). (a) Volmer-Heyrovsky mechanism of HER on 5.9%-HEA (111) and 5.9%-PtNi (111). (b) Volmer-Tafel mechanism of HER on 5.9%-HEA (111) and 5.9%-PtNi (111).

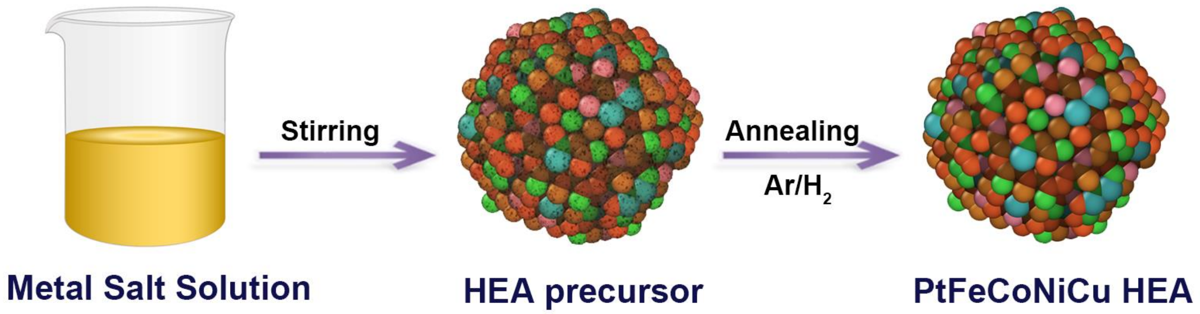

Supplementary Fig. 14. Schematic illustration of the synthesis route of HEA catalysts.

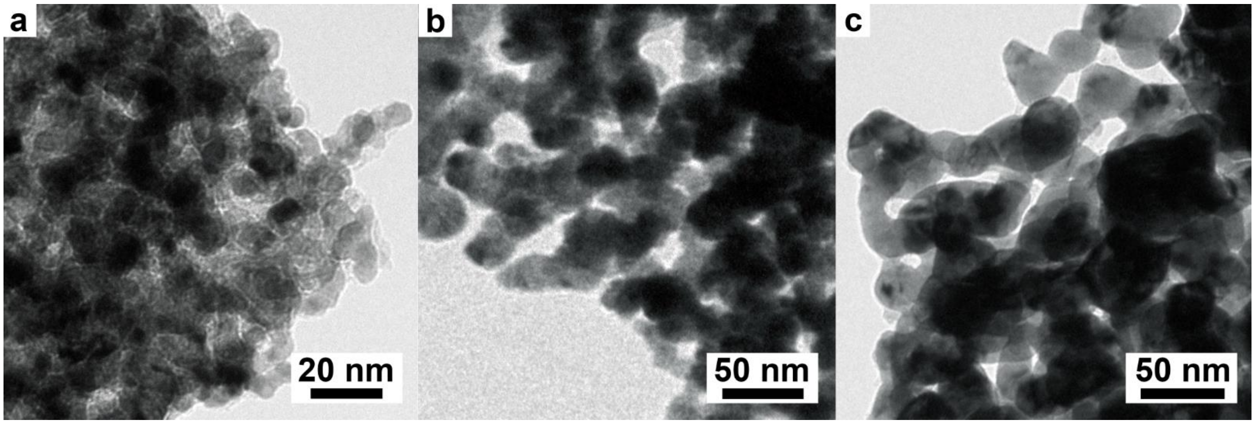

Supplementary Fig. 15. TEM images of (a) HEA-300, (b) HEA-400, and (c) HEA-500.

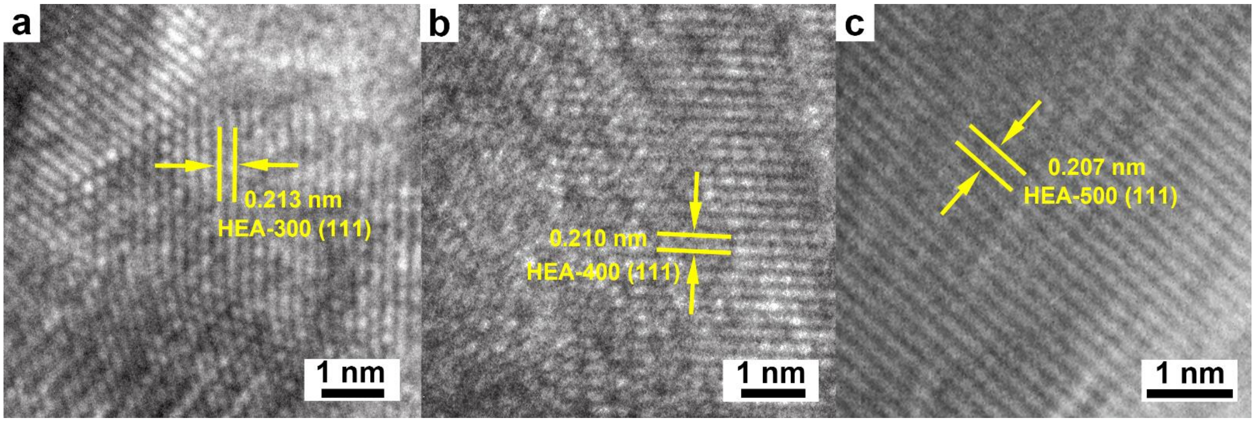

Supplementary Fig. 16. HRTEM images of (a) HEA-300, (b) HEA-400, and (c) HEA-500.

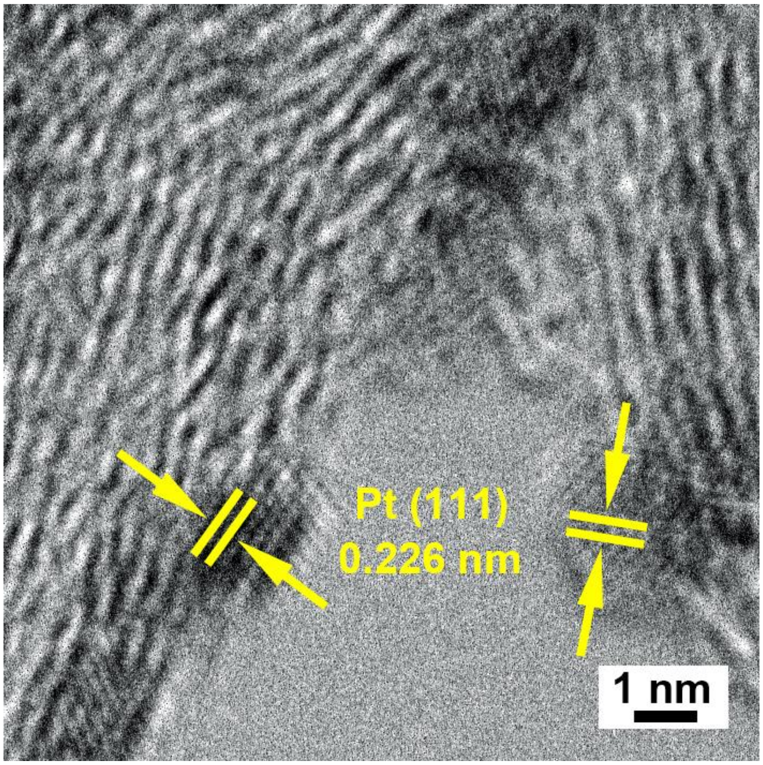

Supplementary Fig. 17. HRTEM image of Pt/C.

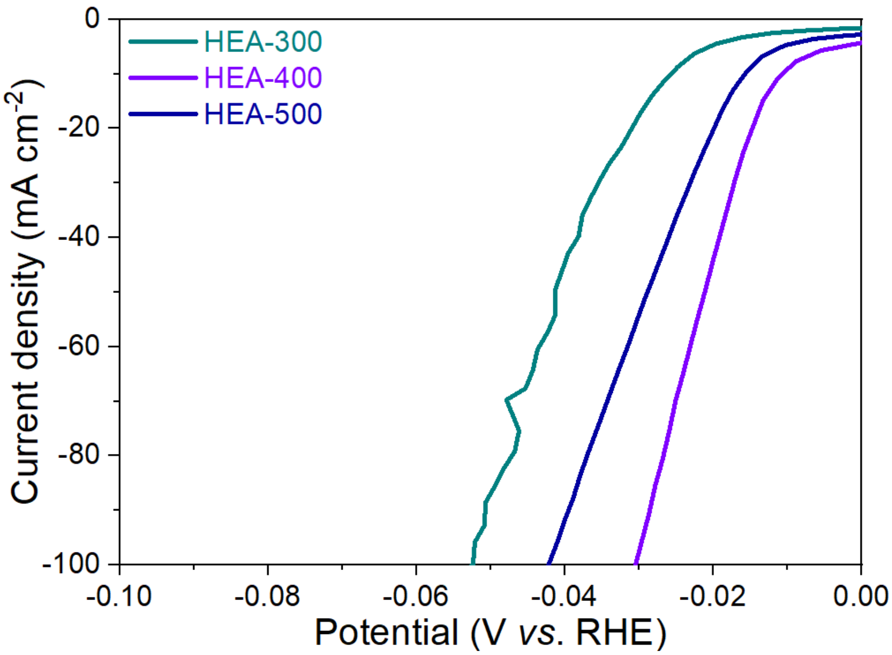

Supplementary Fig. 18. Polarization curves of HEA catalysts annealed at different temperatures.

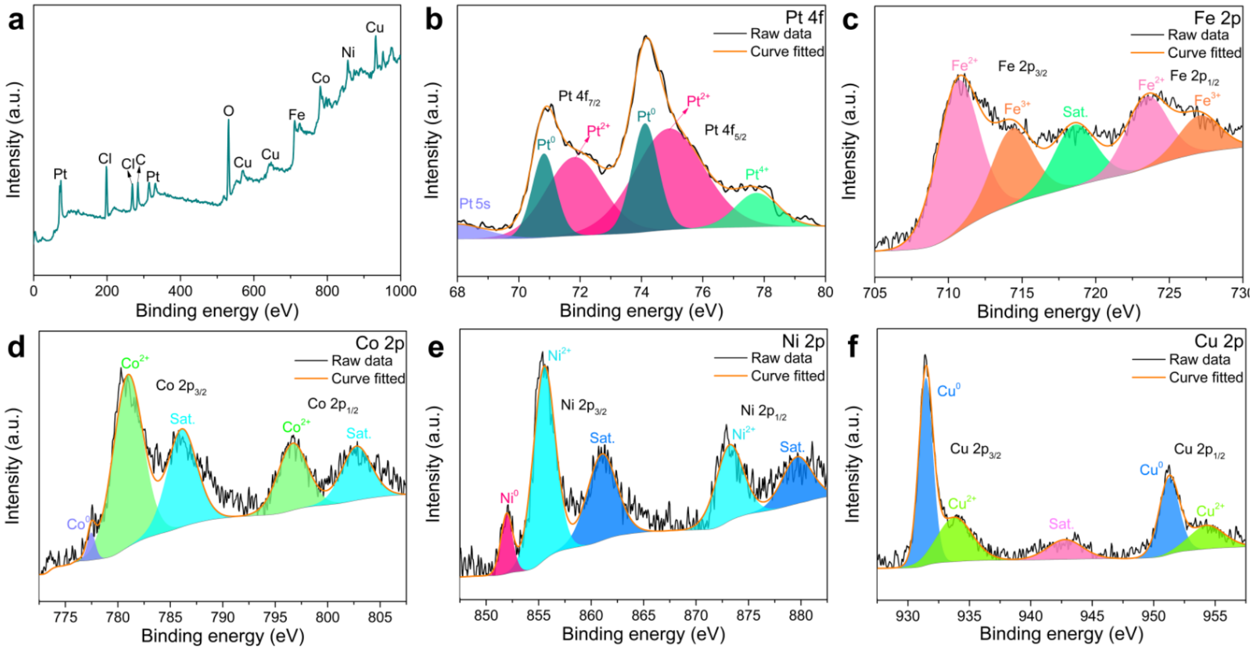

Supplementary Fig. 19. (a) XPS survey spectrum of HEA-300. (b-f) Pt 4f, Fe 2p, Co 2p, Ni 2p and Cu 2p high-resolution XPS spectra of HEA-300.

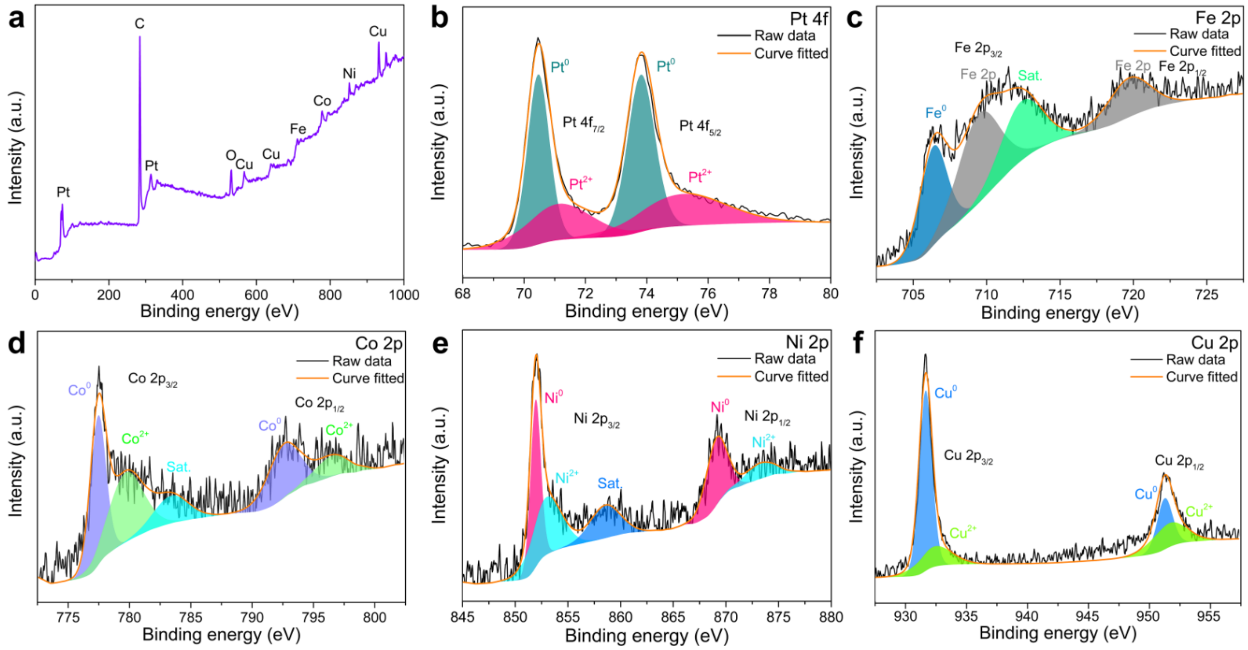

Supplementary Fig. 20. (a) XPS survey spectrum of HEA-400. (b-f) Pt 4f, Fe 2p, Co 2p, Ni 2p and Cu 2p high-resolution XPS spectra of HEA-400.

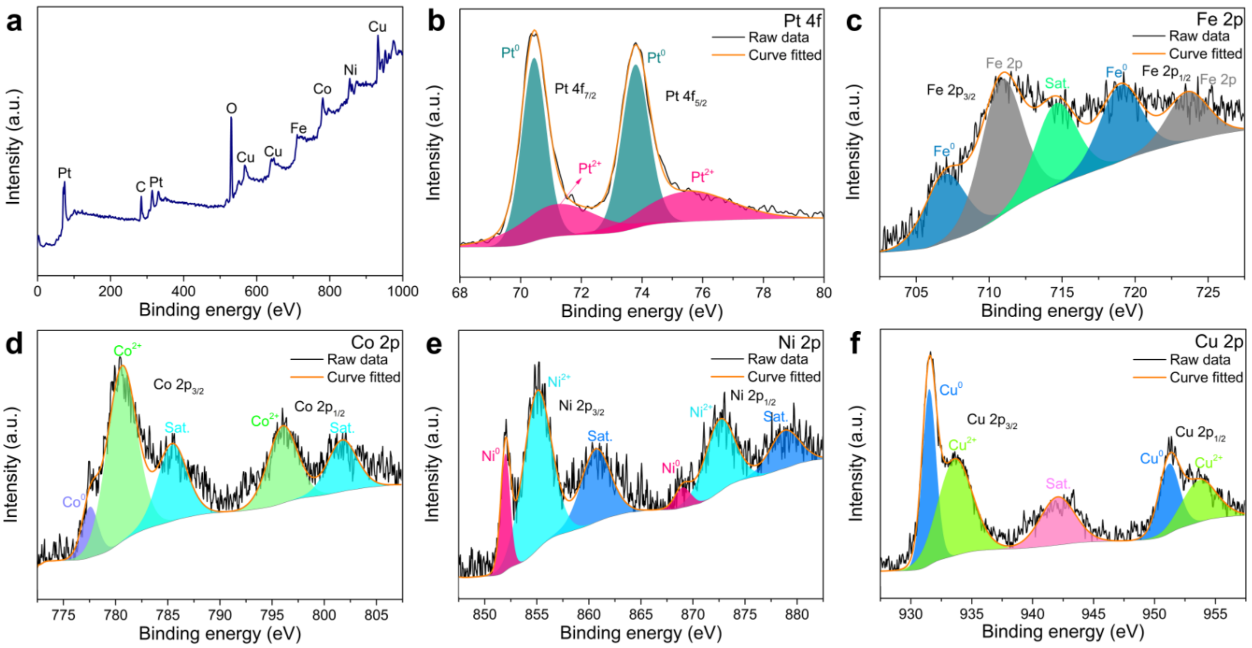

Supplementary Fig. 21. (a) XPS survey spectrum of HEA-500. (b-f) Pt 4f, Fe 2p, Co 2p, Ni 2p and Cu 2p high-resolution XPS spectra of HEA-500.

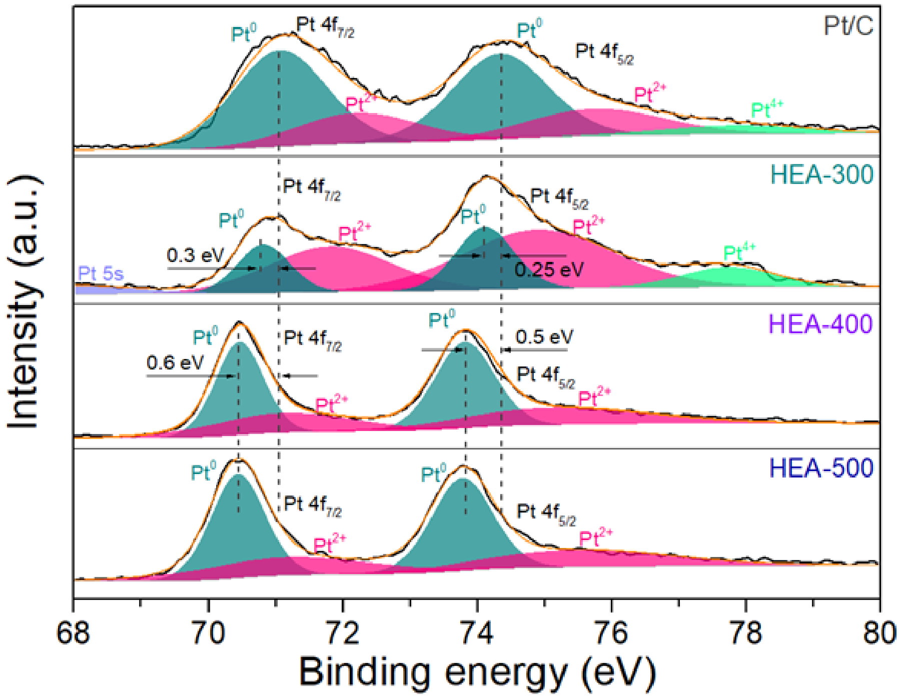

Supplementary Fig. 22. Pt 4f high-resolution XPS spectra of Pt/C, HEA-300, HEA-400, and HEA-500.

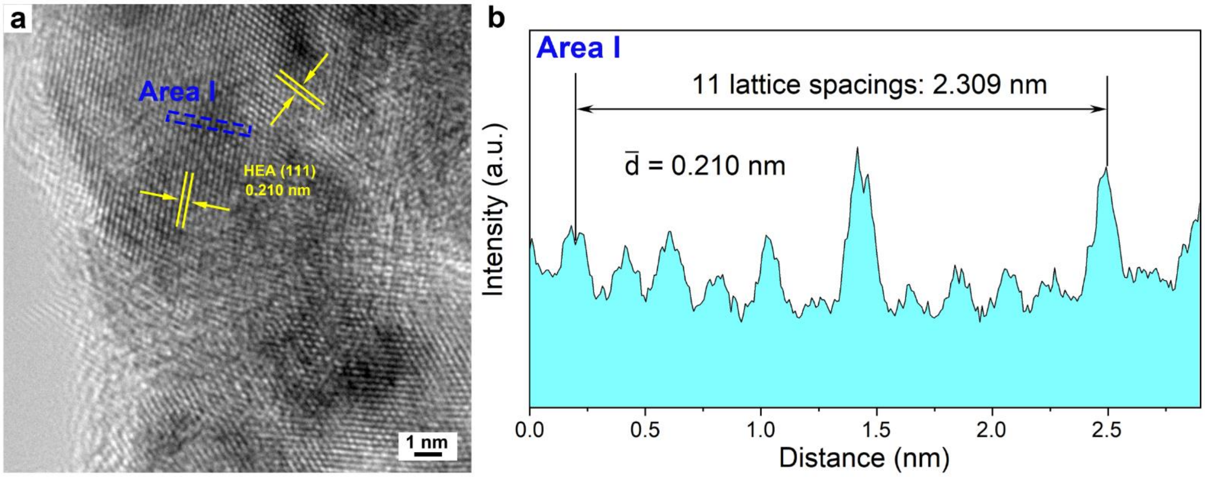

Supplementary Fig. 23. (a) HRTEM image of HEA-400-2000. (b) Intensity line profile of HEA-400-2000 as framed in Area I.

Supplementary Note 1: After 2000 cycles of CV test, HEA-400-2000 presents a (111) lattice spacing of 0.210 nm, corresponding to that of HEA-400 without CV test (see Supplementary Fig. 16b). This means that 2000 cycles of CV test are insufficient to introduce component gradient in HEA-400.

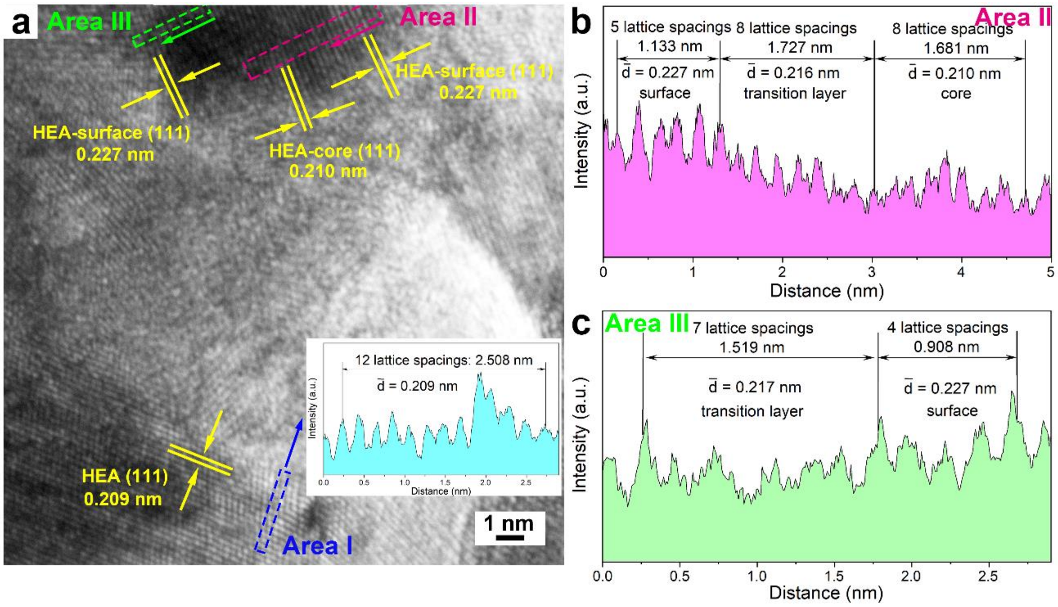

Supplementary Fig. 24. (a) HRTEM image of HEA-400-5000, the inset shows the intensity line profile as framed in Area I. (b) Intensity line profile of HEA-400-5000 as framed in Area II. (c) Intensity line profile of HEA-400-5000 as framed in Area III.

Supplementary Note 2: After 5000 cycles of CV test, besides the original lattice spacing of HEA-400 (0.209 nm, see Area I in Supplementary Fig. 24a), the lattice spacings of activated surface can also be observed, as shown in Area II and Area III in Supplementary Fig. 24a. As shown in Supplementary Fig. 24b, in the activated HEA-surface, the average (111) lattice spacing is 0.227 nm, corresponding to that of Pt (111) lattice. It means that Pt element dominates in the activated surface layer of HEA-400-5000. In the transition layer, the average (111) lattice spacing is 0.216 nm, which is attributed to the partial etching of non-Pt elements. In the core of activated HEA-400-5000, the average (111) lattice spacing is 0.210 nm, which corresponds to that of HEA-400 without CV test, indicating that the etching effect only appears in the outermost layers of HEA. This is also the reason for the observed lattice spacing of 0.209 nm which belongs to the original HEA-400 (as framed in Area I). The gradually decreasing average lattice spacing from the surface to the core demonstrates that the component gradient exists in HEA-400-5000. Similarly in Area III, the average lattice spacings of HEA-surface and HEA-core are 0.227 and 0.217 nm, respectively, which further supports the existence of component gradient in HEA-400-5000.

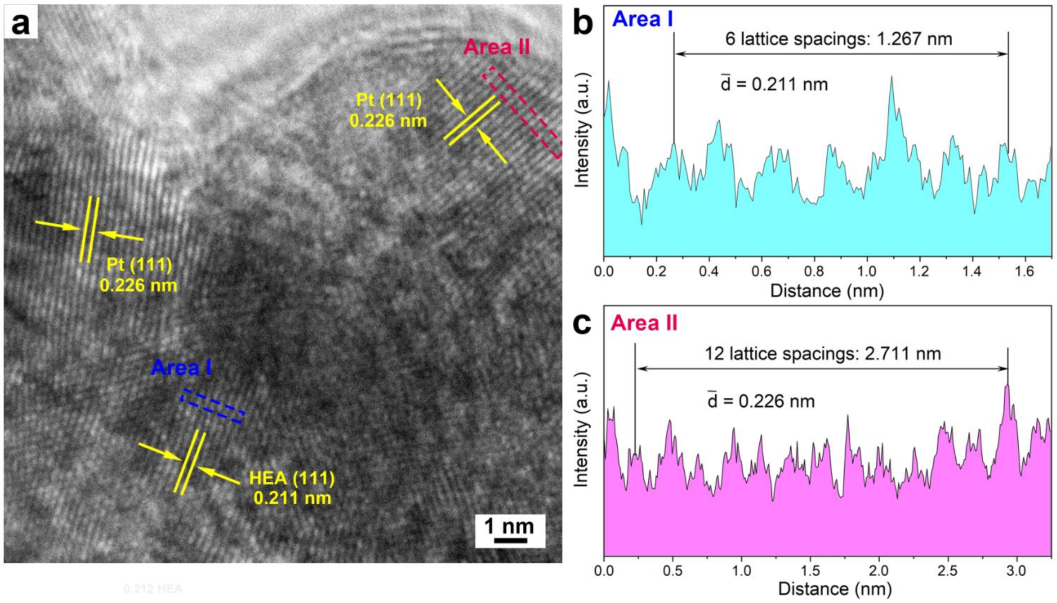

Supplementary Fig. 25. (a) HRTEM image of HEA-400-10000. (b) Intensity line profile of HEA-400-10000 as framed in Area II. (c) Intensity line profile of HEA-400-10000 as framed in Area II.

Supplementary Note 3: After 10000 cycles of CV test, HEA-400-10000 presents the lattice spacings of 0.211 nm for HEA (111) and 0.226 nm for Pt (111), respectively. This result indicates that extra CV tests will lead to excessive etching of non-noble elements in HEA-surface, which will reduce the component gradient.

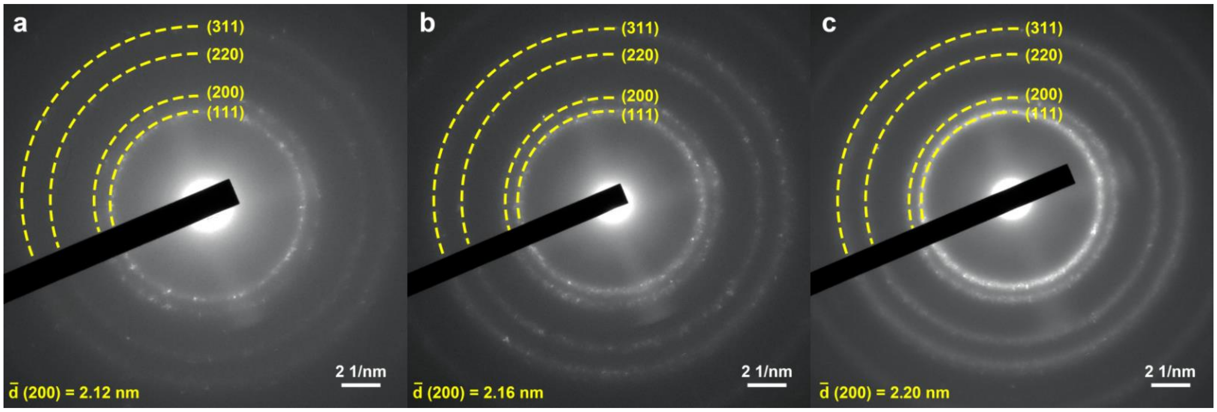

Supplementary Fig. 26. SAED patterns of (a) HEA-400-2000, (b) HEA-400-5000, and (c) HEA-400-10000.

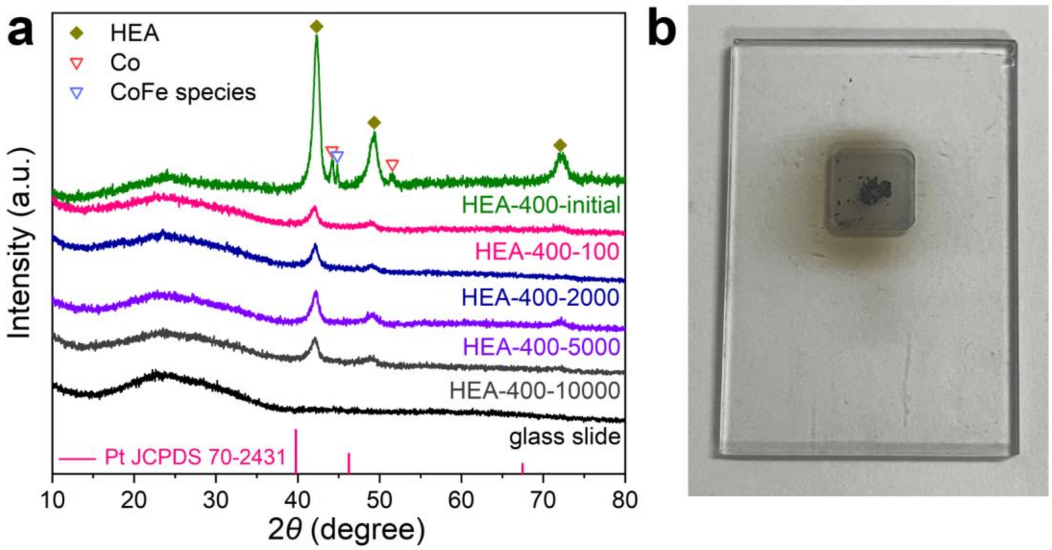

Supplementary Fig. 27. (a) XRD patterns of HEA-400-initial, HEA-400-100, HEA-400-2000, HEA-400-5000 and HEA-400-10000. (b) Photograph of CV-activated HEA-400 catalysts covered on a glass slide, which is used for XRD characterization.

Supplementary Note 4: HEA-400-initial presents three main peaks at 42.3°, 49.3° and 72.1°, corresponding to the (111), (200) and (220) planes of HEA. The peaks detected at 44.2° and 51.5° correspond to the (111) and (200) planes of Co (JCPDS 89-7093), the peak detected at 44.8° corresponds to the (110) plane of CoFe species (maybe CoFe, JCPDS 44-1433 or Co_3Fe_7 JCPDS 48-1816). After 100 cycles of CV test, all the peaks of Co or CoFe species disappear, indicating that HEA-400-100 is a well-defined HEA. The Co and CoFe species that fail to form HEA are etched first during the CV process. In addition to HEA-400-100, HEA-400-2000, HEA-400-5000 and HEA-400-10000 all present well-defined HEA peaks. Although small Pt nanoparticles have been formed in HEA-400-10000 (see Supplementary Fig. 25), no Pt characterization peaks are detected in HEA-400-10000, which is attributed to the small amount of the formed Pt particles compared to the whole HEA. The broad peaks detected at ~ 23.4° in HEA catalysts correspond to the glass slide. The appearance of these peaks is because that the amounts of samples collected after HER testing is small, which is not sufficient to fully cover the glass slide, as shown in Supplementary Fig. 27b.

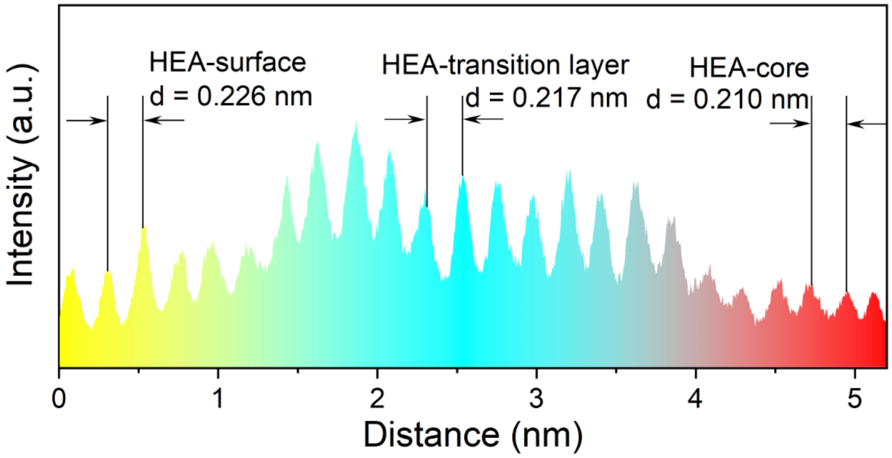

Supplementary Fig. 28. Intensity line profile from the surface to the core in HEA-400-5000 as framed in Fig. 3c. The marked 3 lattice spacings correspond to the HEA-surface, HEA-transition layer and HEA-core in Fig. 3c.

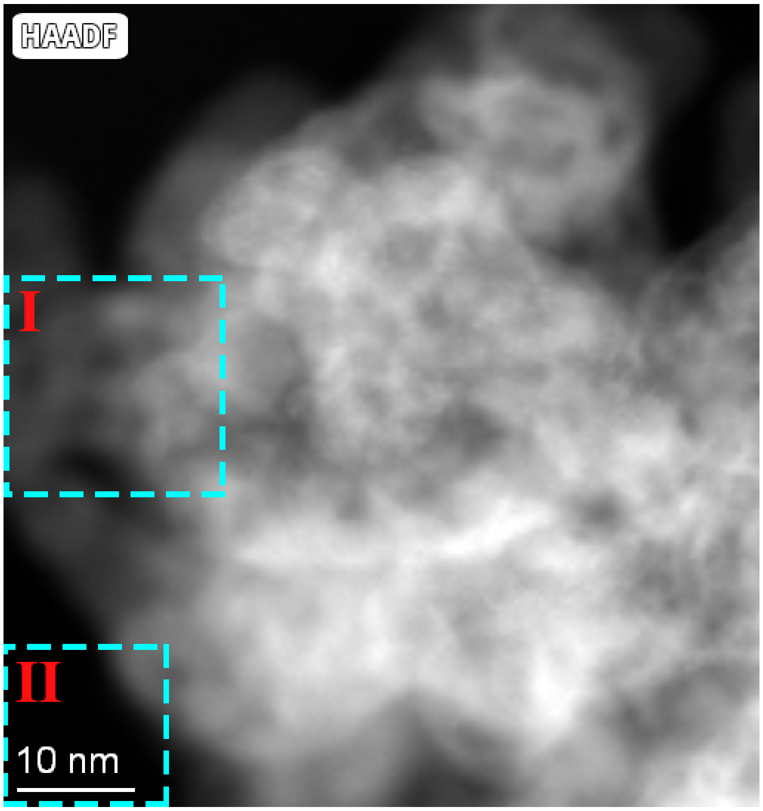

Supplementary Fig 29. Origin HAADF TEM image of Fig. 3e. The circled areas I and II are selected for detailed analysis, as shown in Supplementary Figs. 30-31, respectively.

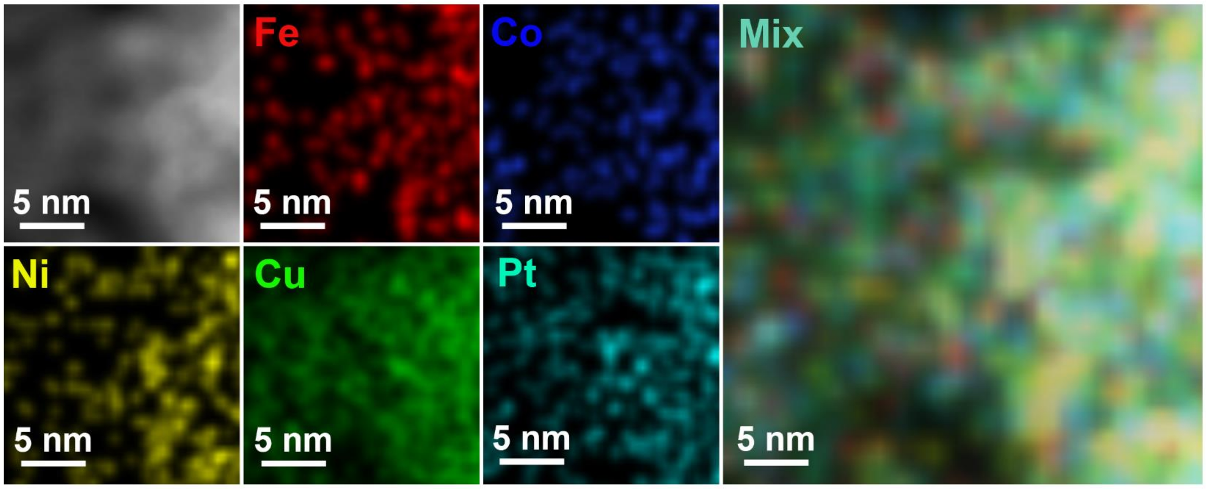

Supplementary Fig. 30. HAADF-EDS elemental maps of HEA-400-5000 selected in the near-surface area I in Supplementary Fig. 29.

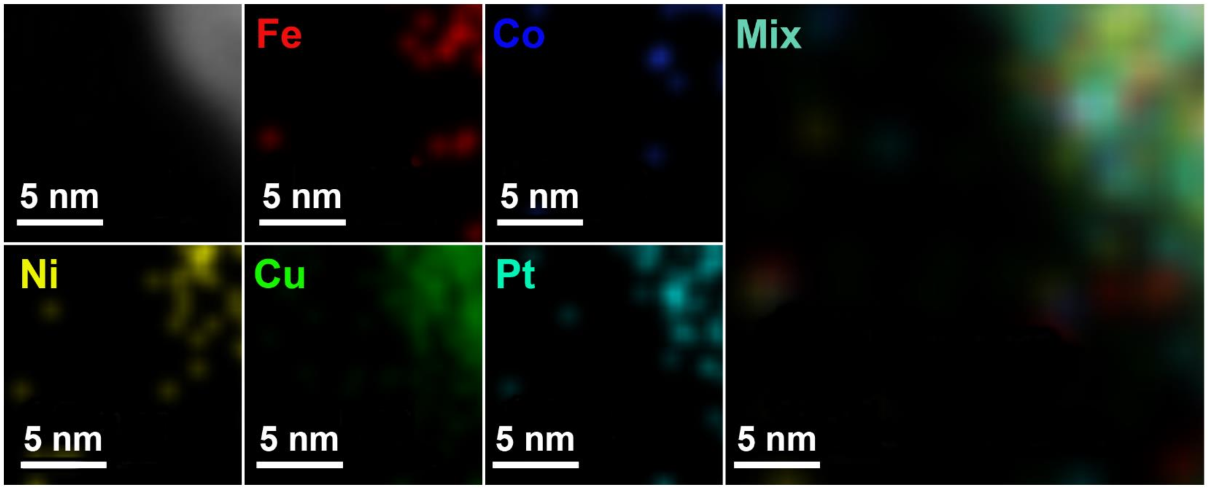

Supplementary Fig. 31. HAADF-EDS elemental maps of HEA-400-5000 selected in the near-surface area II in Supplementary Fig. 29.

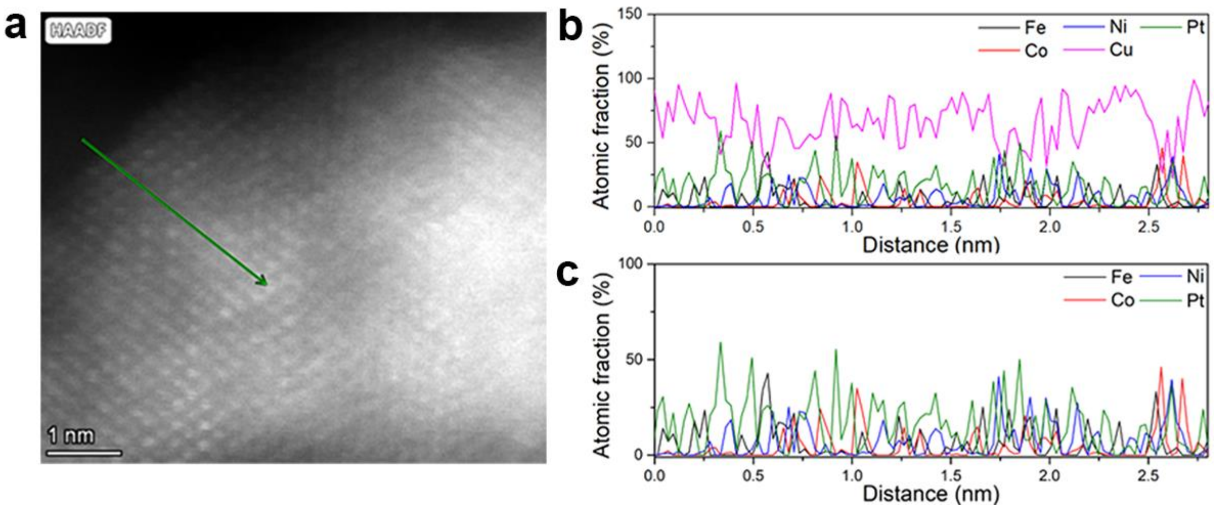

Supplementary Fig. 32. (a) HAADF image of HEA-400-5000. (b) HAADF line scan of Fe, Co, Ni, Cu, and Pt elements along the green arrow shown in Supplementary Fig. 32a. (c) HAADF line scan of Fe, Co, Ni, and Pt elements along the green line shown in Supplementary Fig. 32a. Note that inevitable errors exist in the data of the Cu element due to the equipment factors. Thus, they are removed for clarity.

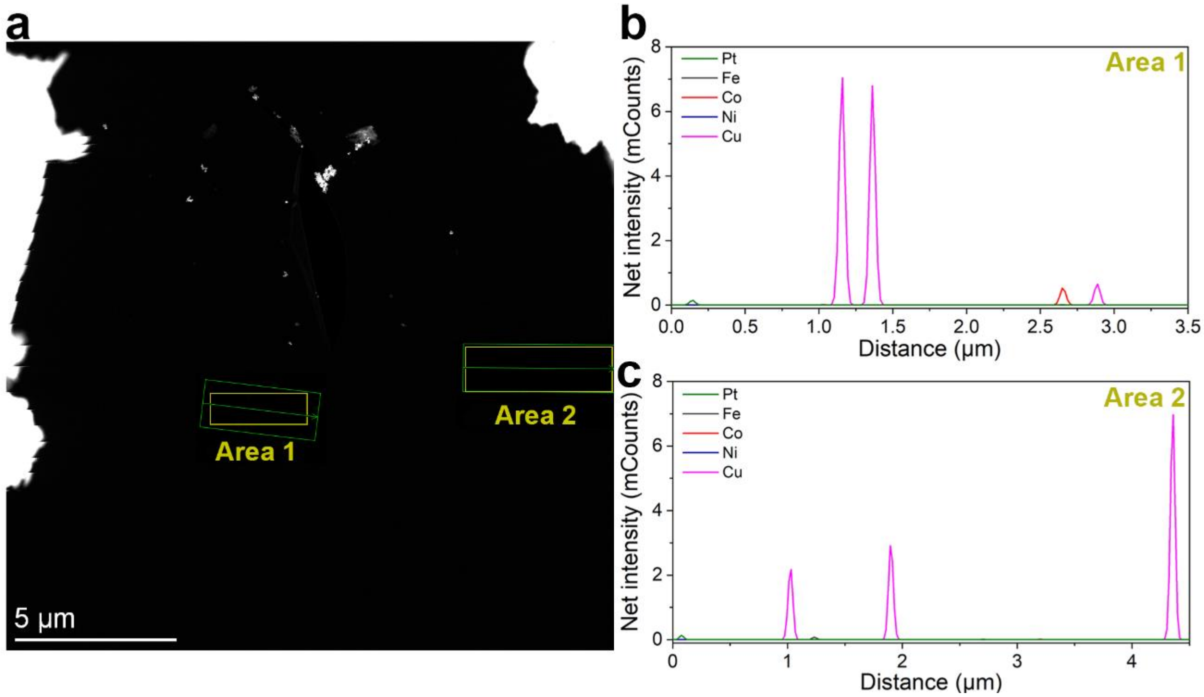

Supplementary Fig. 33. (a) HAADF image of HEA-400-5000. The white areas are the HEA particles, the black area is blank. (b) HAADF line scan of Fe, Co, Ni, Cu, and Pt elements along the green arrow shown in Area 1. (c) HAADF line scan of Fe, Co, Ni, Cu, and Pt elements along the green arrow shown in Area 2.

Supplementary Note 5: The excessive Cu atomic fraction in Supplementary Fig. 32b derives from the equipment factors during the HAADF-STEM characterization. To conduct the HAADF-STEM characterization, the sample needs to be pretreated through dispersing on a Mo support film, which is held by a copper specimen holder. The holder is then sent into a vacuum environment for the characterization. In the vacuum environment, the EDS detection signals are easily affected by the surrounding environment. Due to the presence of Cu element in the specimen holder, the detected Cu element content in the sample will inevitably be higher than the actual situation. To support this issue, HAADF line scan characterizations of the blank area without samples are conducted. As shown in Supplementary Fig. 33, a large number of Cu elements are detected due to the equipment factors, even if there are no samples.

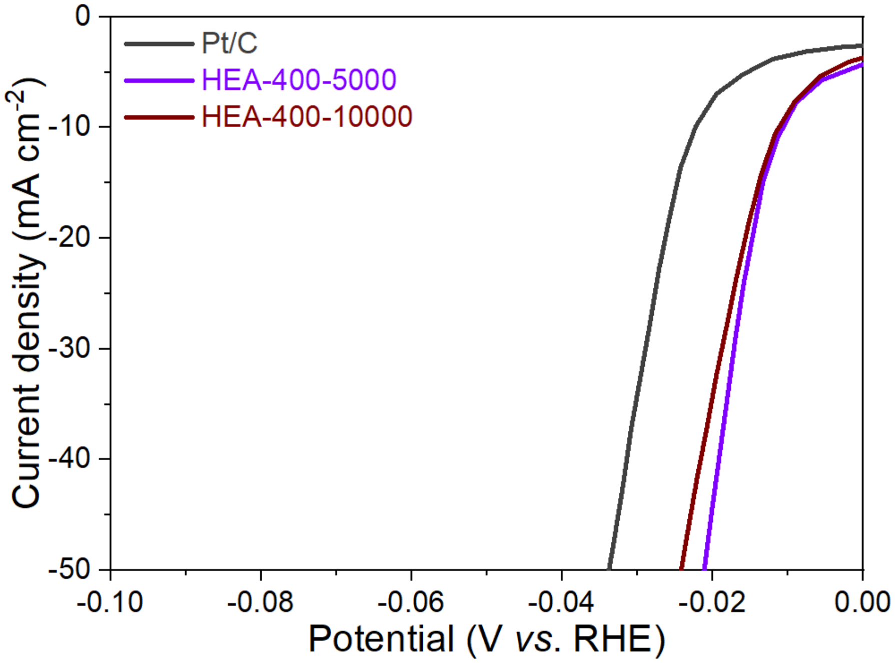

Supplementary Fig. 34. Polarization curves of HEA-400-5000, HEA-400-10000 and Pt/C.

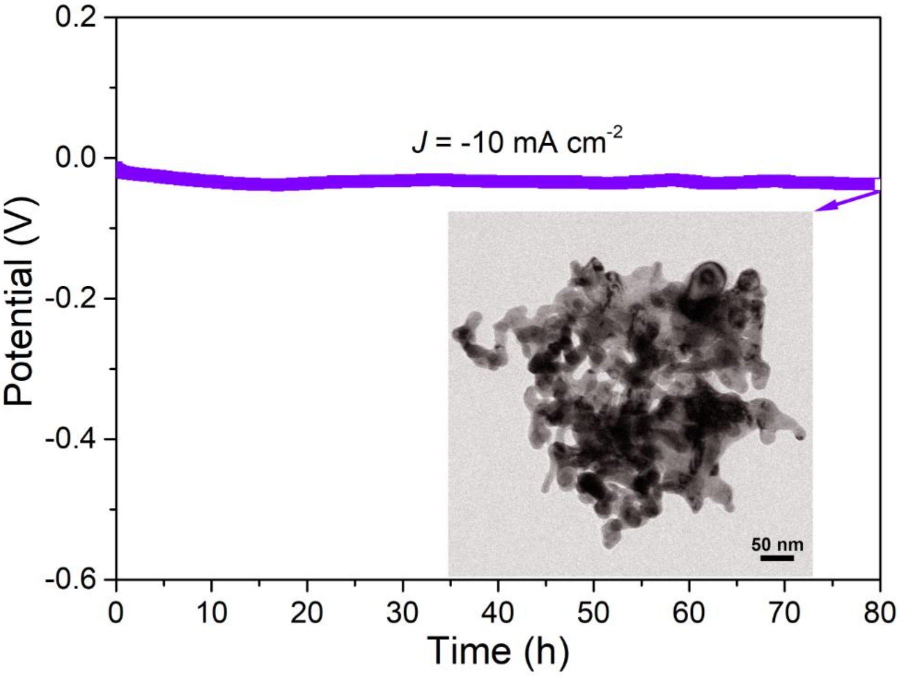

Supplementary Fig. 35. The galvanostatic plot of activated HEA-400 (HEA-400-5000) at -10 mA cm^{-2} for 80 h. The inset shows a TEM image of HEA-400-5000 after the 80-h test.

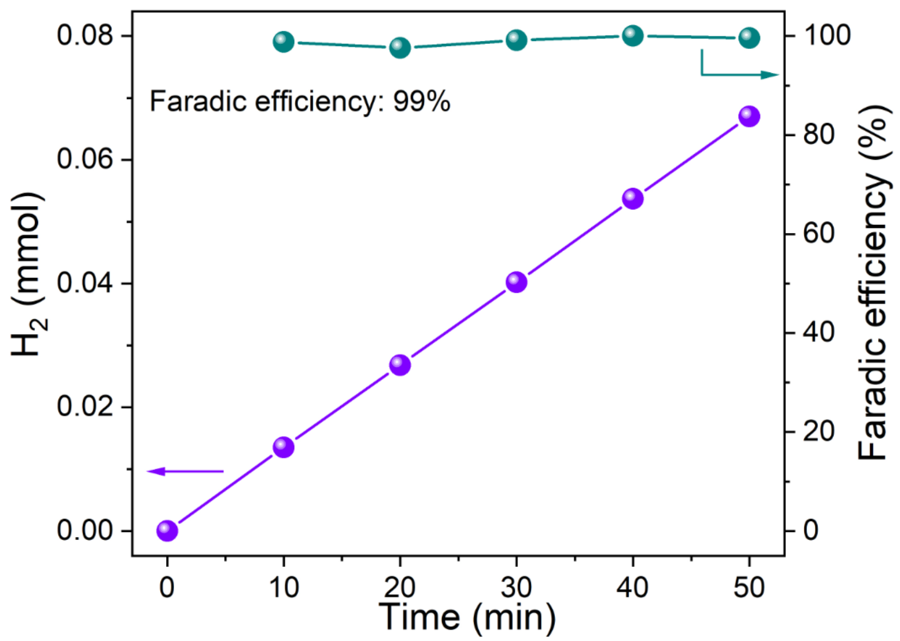

Supplementary Fig. 36. Hydrogen production and the corresponding faradic efficiency for HEA-400-5000.

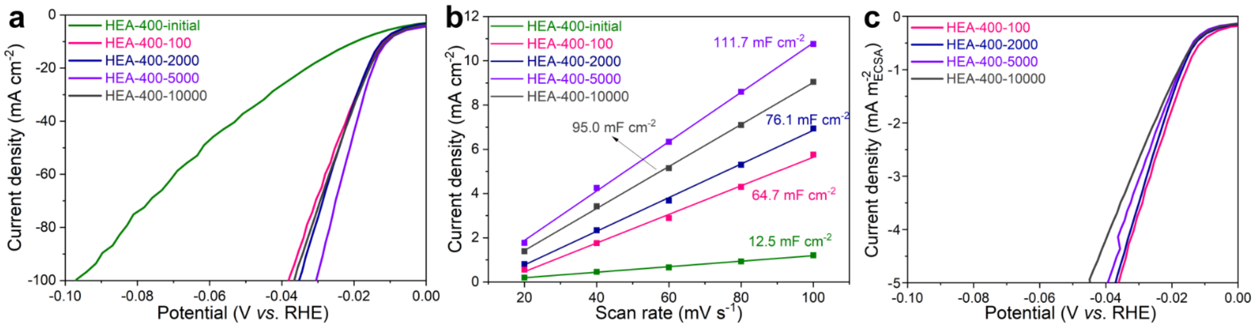

Supplementary Fig. 37. (a) Polarization curves of HEA-400 with different cycles of CV activation. (b) Plots of capacitive currents with various scan rates for HEA-400 with different cycles of CV activation. (c) Polarization curves normalized to ECSA for HEA-400 with different cycles of CV activation.

Supplementary Note 6: For HEA-400, the C_{dl} increases continuously with the CV progress and reaches the maximum value of 111.7 mF cm^{-2} after 5000 CV cycles (see Supplementary Fig. 37b). The greatly increased C_{dl} derives from the gradually increasing exposed Pt sites and the enlarged component gradient during the CV test. Further CV tests will reduce the C_{dl} of HEA-400 (the C_{dl} of HEA-400-10000 is 95.0 mF cm^{-2}), which may be attributed to the diminished component gradient in the surface after excessive CV tests. Corresponding to the C_{dl}, the HER activity of HEA-400 continues to improve with the CV progress and HEA-400-5000 shows the best performance with an η_{100} of 30.7 mV (see Supplementary Fig. 37a). Further CV tests will reduce the HER activity of HER-400, and the HEA-400-10000 sample shows an increased η_{100} of 36.6 mV. When normalized to the ECSA, the specific activity of HEA-400 declines slightly with the CV progress. This is because the electron gradient in HEA surface gradually decreases during the CV progress.

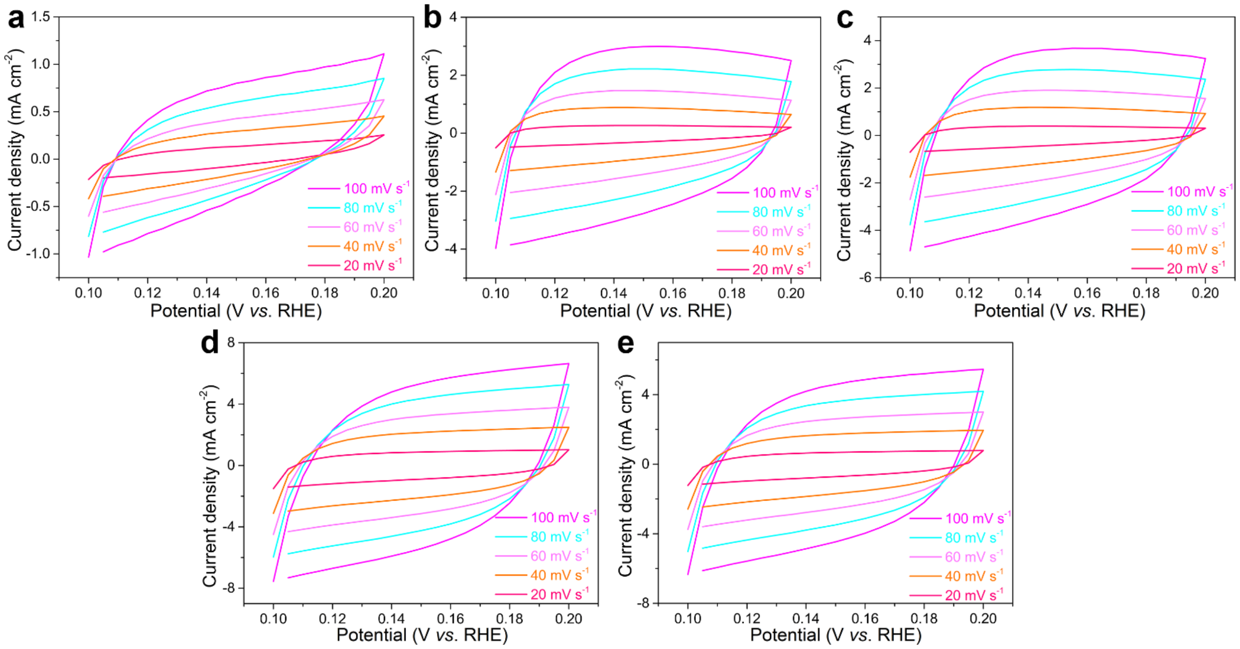

Supplementary Fig. 38. (a-e) Detailed cyclic voltammetry data for HEA-400-initial, HEA-400-100, HEA-400-2000, HEA-400-5000 and HEA-400-10000 to determine the double layer capacitance, respectively.

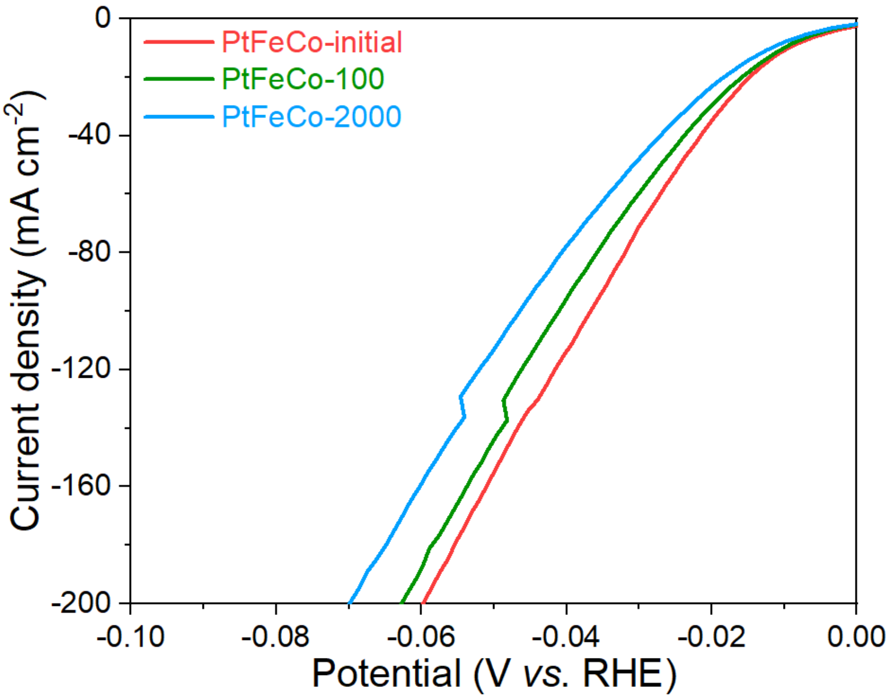

Supplementary Fig. 39. Polarization curves of a ternary PtFeCo alloy before and after CV test. The electrochemical activation effect is not observed in PtFeCo, demonstrating that the dual-gradient system is unable to form when the components in the alloy are inadequate.

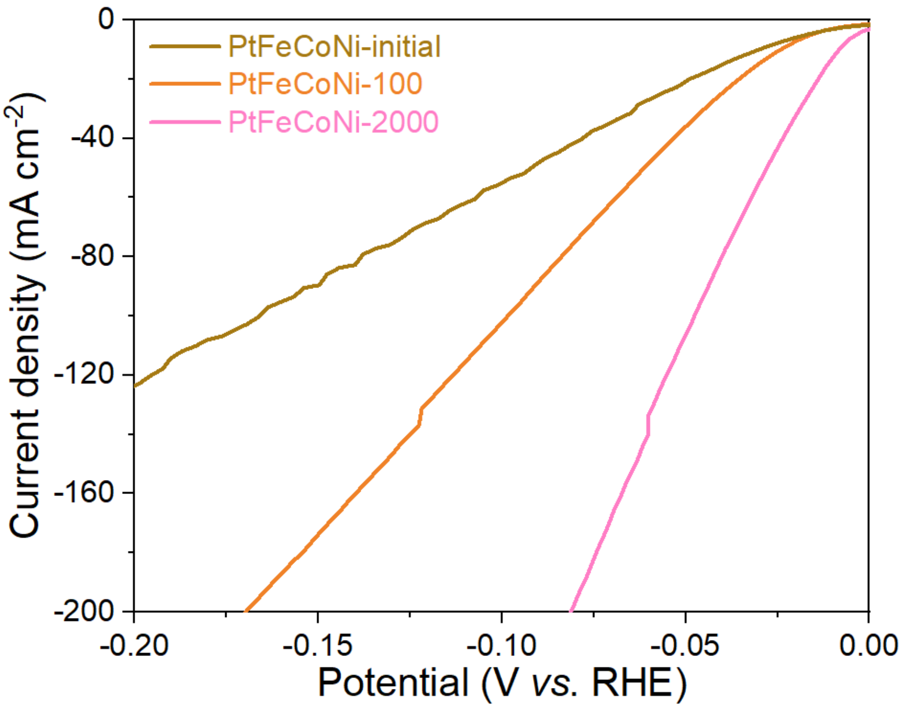

Supplementary Fig. 40. Polarization curves of a quaternary PtFeCoNi alloy before and after CV test. The electrochemical activation effect is observed.

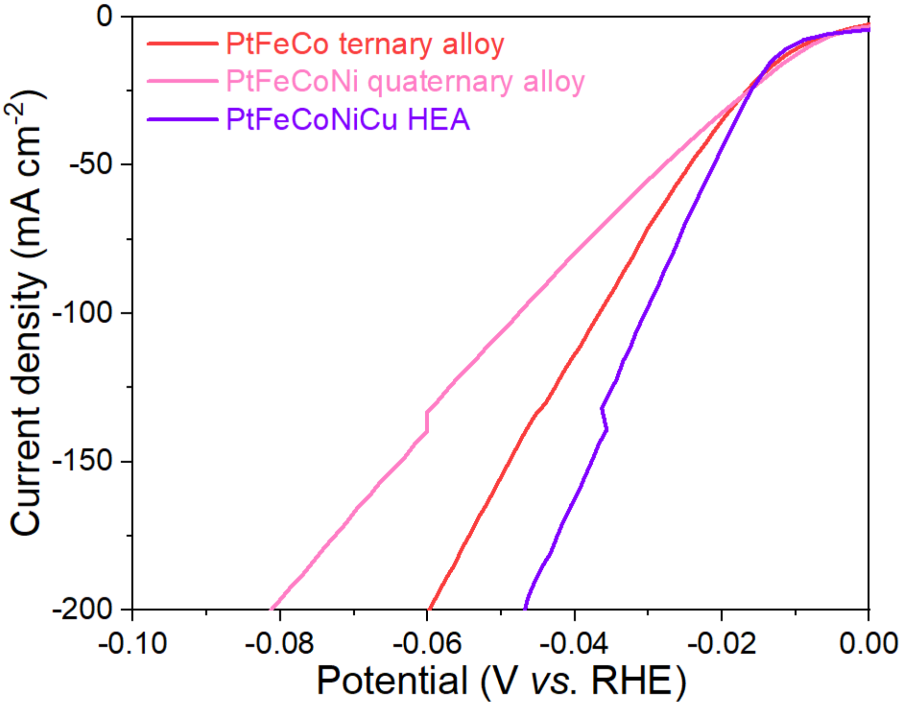

Supplementary Fig. 41. Comparison of polarization curves among PtFeCo ternary alloy (PtFeCo-initial), PtFeCoNi quaternary alloy (PtFeCoNi-2000) and PtFeCoNiCu HEA (HEA-400-5000).

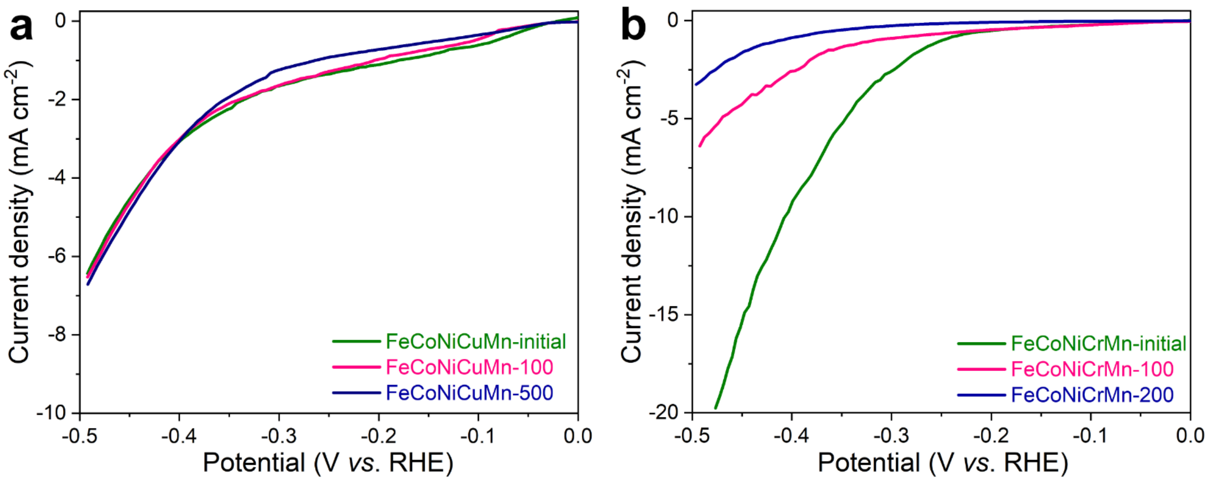

Supplementary Fig. 42. (a) Polarization curves of a FeCoNiCuMn HEA with different cycles of CV test. (b) Polarization curves of a FeCoNiCrMn HEA with different cycles of CV test.

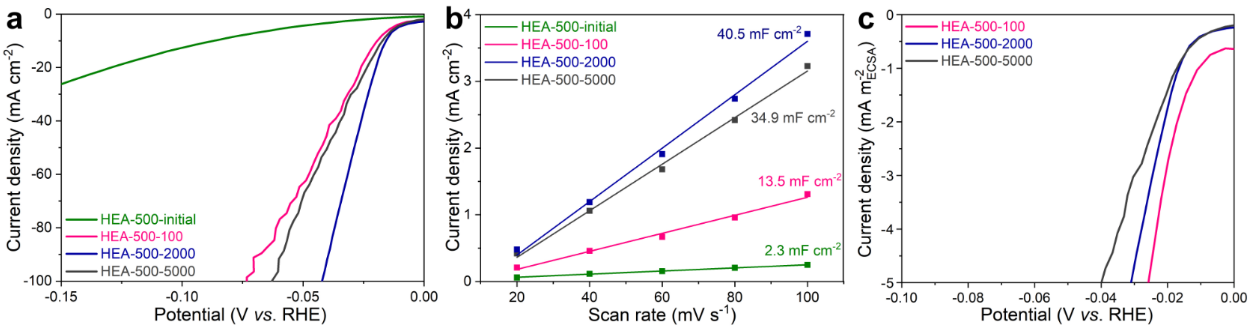

Supplementary Fig. 43. (a) Polarization curves of HEA-500 with different cycles of CV activation. (b) Plots of capacitive currents with various scan rates for HEA-500 with different cycles of CV activation. (c) Polarization curves normalized to ECSA for HEA-500 with different cycles of CV activation.

Supplementary Note 7: For HEA-500, the C_{dl} and HER activity increase continuously with the CV progress and reach the best performance after 2000 CV cycles. The HEA-500-2000 shows a C_{dl} of 40.5 mF cm^{-2} and an η_{100} of 42.1 mV. Further CV tests will reduce the C_{dl} and HER activity of HEA-500. HEA-500-5000 shows a diminished C_{dl} of 34.9 mF cm^{-2} and an increased η_{100} of 62.5 mV. When normalized to the ECSA, the specific activity of HEA-500 declines slightly with the CV progress, which is also attributed to the diminished electron gradient in the surface.

Supplementary Note 8: The trends of HER performance change during the CV test for HEA-400 and HEA-500 are the same. Both of them experience a significant increase followed by a slight decrease in HER performance with continuous CV test. However, the cycles of CV tests to bring the best HER performance for HEA-400 and HEA-500 are different. During the CV activation process, the HEA particles undergo the removal of impurities (as confirmed in the XRD patterns shown in Supplementary Fig. 27a) and the formation of component gradient. More impure phases exist in HEA-400 than HEA-500. Therefore, more CV tests are performed to etch these impurities in HEA-400 before bringing the component gradient on the surface. Consequently, more activation cycles are performed to bring the best HER performance for HEA-400 than HEA-500.

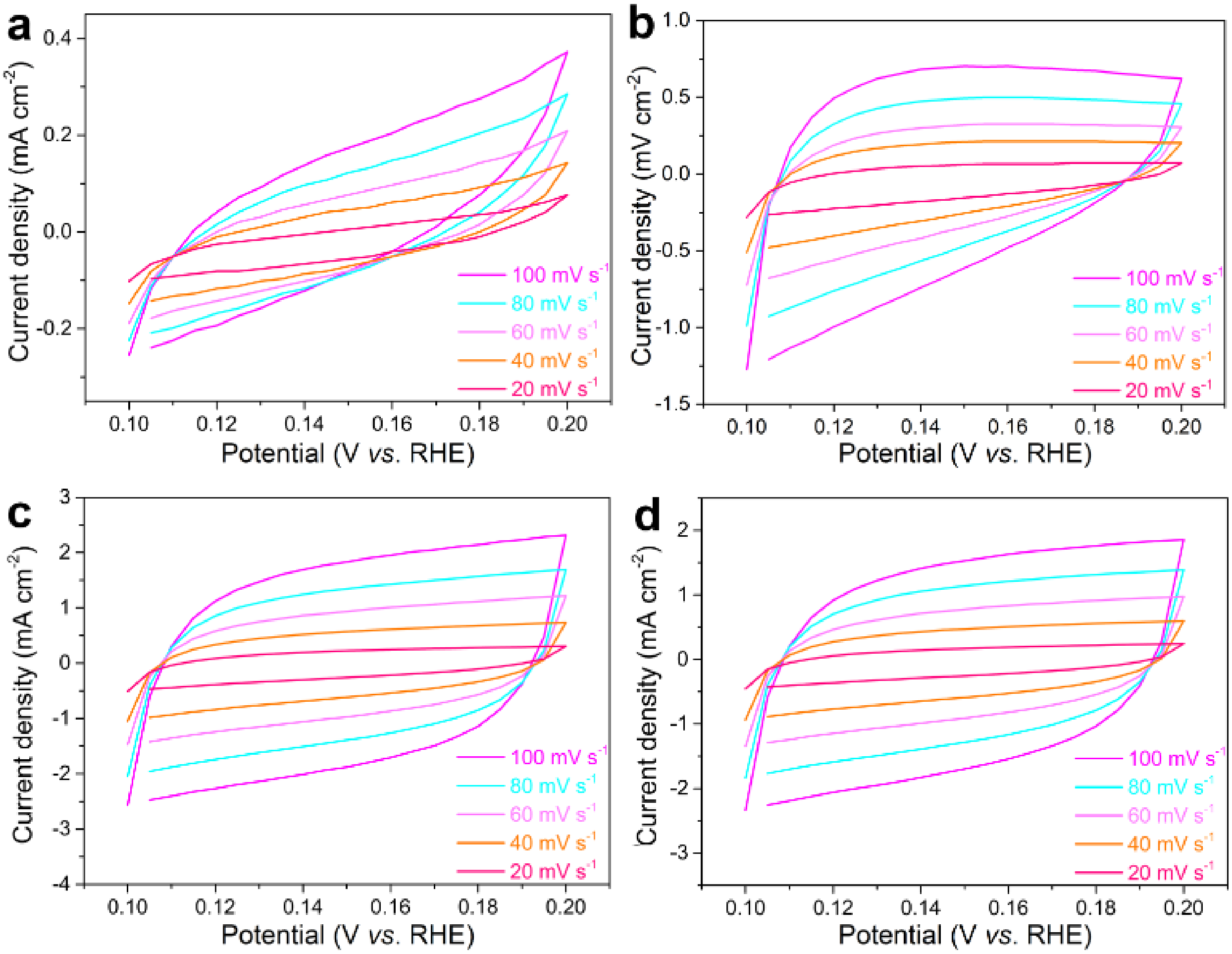

Supplementary Fig. 44. (a-d) Detailed cyclic voltammetry data for HEA-500-initial, HEA-500-100, HEA-500-2000, and HEA-500-5000 to determine the double layer capacitance, respectively.

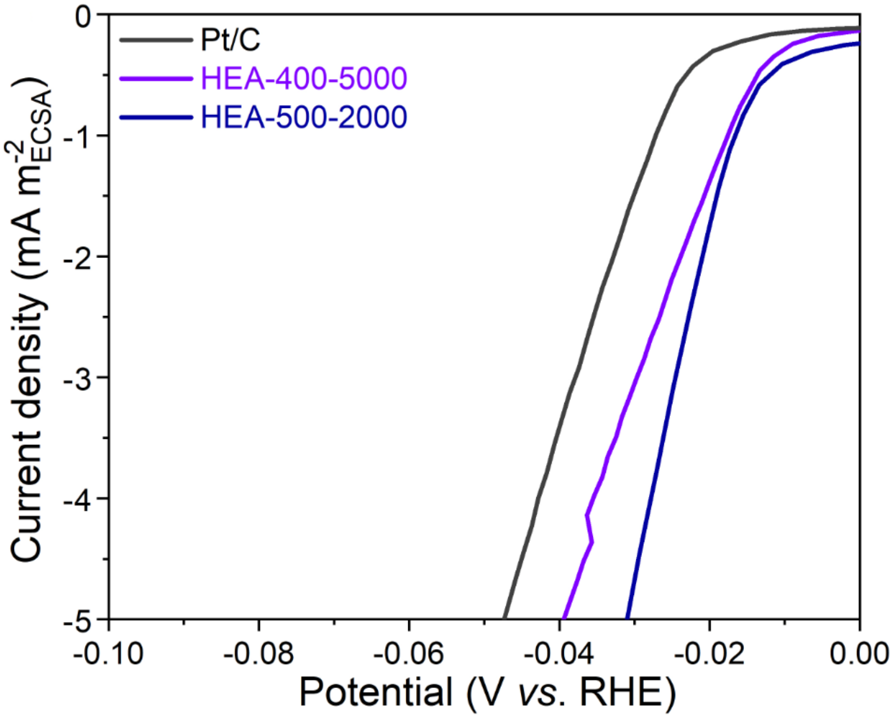

Supplementary Fig. 45. Polarization curves normalized to ECSA of HEA-400-5000, HEA-500-2000, and Pt/C.

Supplementary Note 9: The activated HEA-400 and HEA-500 present superior specific activity than Pt/C. This is because HEA-400-5000 and HEA-500-2000 still possess stronger surface electron gradients than Pt/C. Combined with the component gradient formed during the CV progress, the superior HER activity of the activated HEA than Pt/C derives from the dual gradients catalytic system.

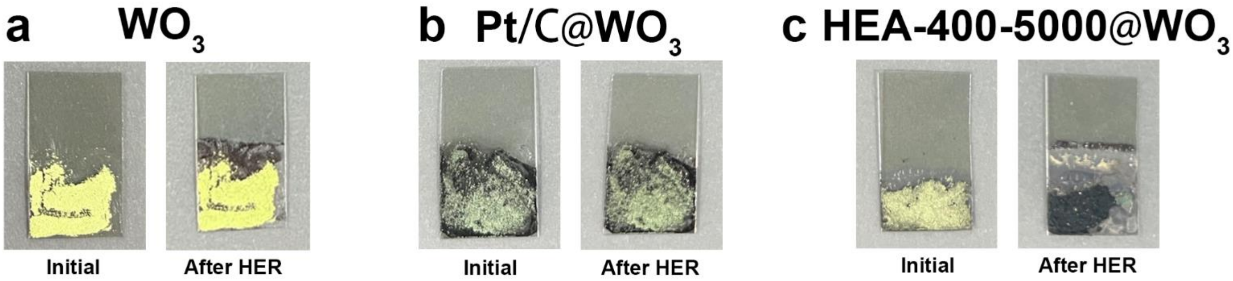

Supplementary Fig. 46. Color change photographs of (a) WO_3, (b) mixture of Pt/C@WO_3 and (c) mixture of HEA-400-5000@WO_3. The photographs are taken before and after the HER process as shown in Supplementary Fig. 47.

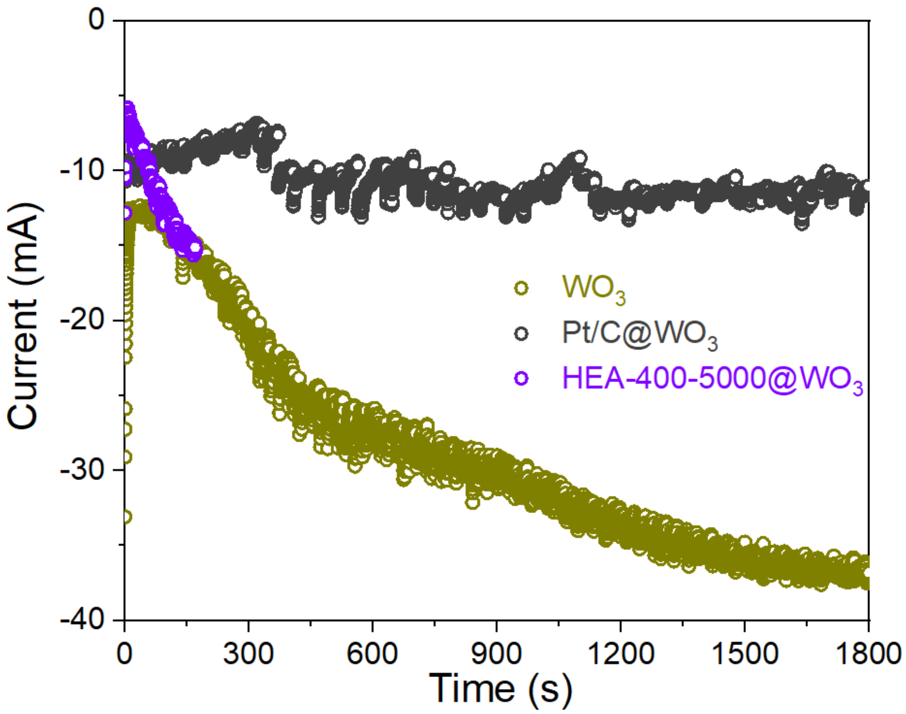

Supplementary Fig. 47. HER galvanostatic plots for WO_3, Pt/C@WO_3 and HEA-400-5000@WO_3 to identify the spillover mechanism.

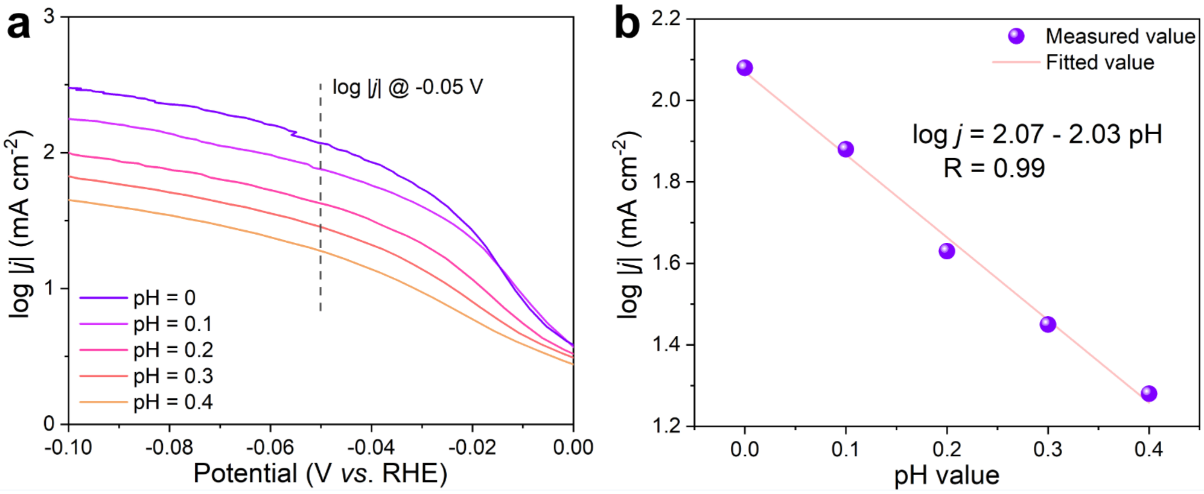

Supplementary Fig. 48. (a) Tafel curves of HEA-400-5000 in H_2SO_4 with pH ranging from 0 to 0.4. (b) The liner plot of log |*j*| at -0.05 V (*vs*. RHE) *vs*. pH for HEA-400-5000.

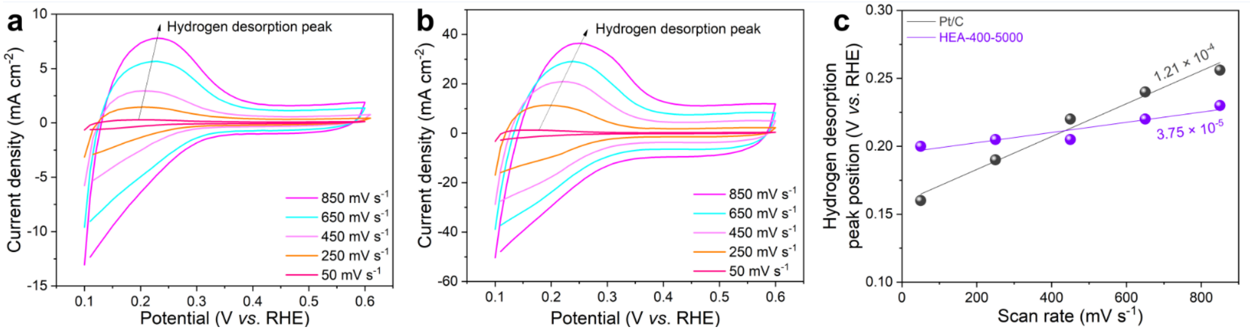

Supplementary Fig. 49. (a) CV profiles of HEA-400-5000 with the scan rate from 65 to 850 mV s^{-1} in 0.5 M H_2SO_4. (b) CV profiles of Pt/C with the scan rate from 65 to 850 mV s^{-1} in 0.5 M H_2SO_4. (c) Plots of hydrogen desorption peak position *vs*. scan rates for HEA-400-5000 and Pt/C.

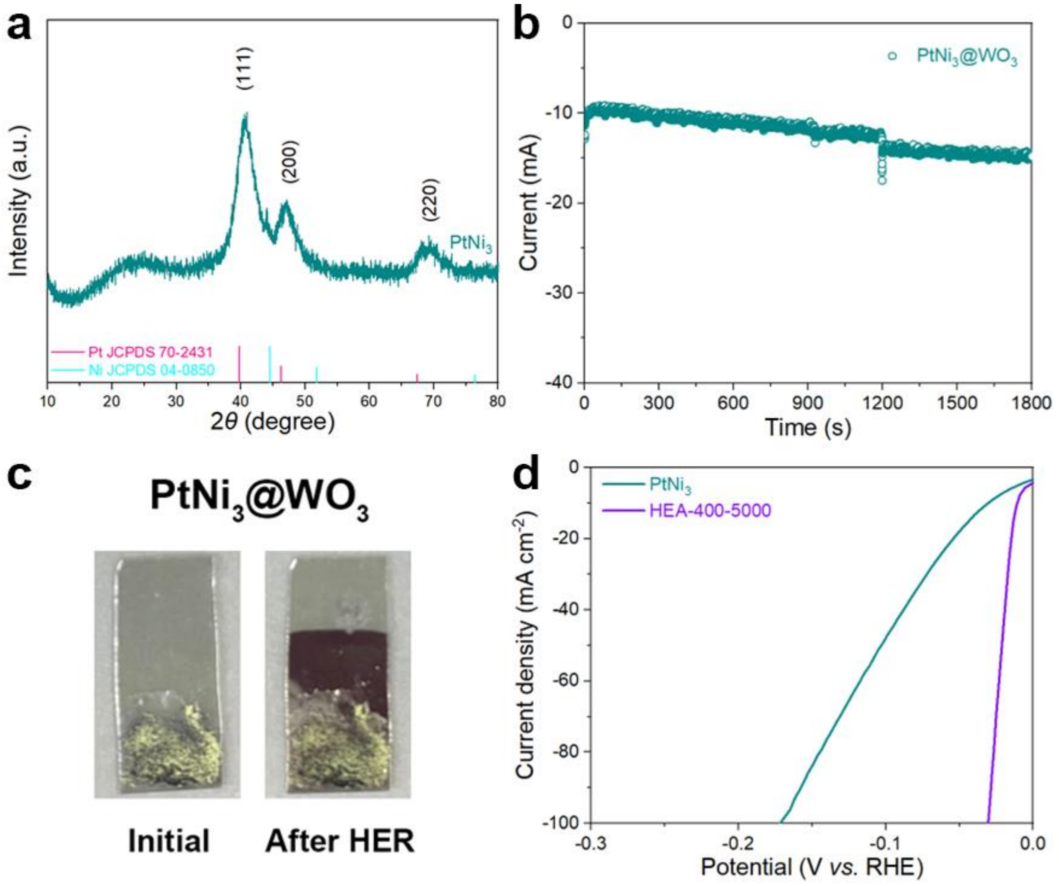

Supplementary Fig. 50. (a) XRD patterns of $PtNi_3$. (b) HER galvanostatic plots for mixture of $PtNi_3@WO_3$ to identify the spillover mechanism. (c) Color change photographs of $PtNi_3@WO_3$. The photographs are taken before and after the HER process as shown in Supplementary Fig. 50b. (d) Polarization curves of HEA-400-5000 and $PtNi_3$.

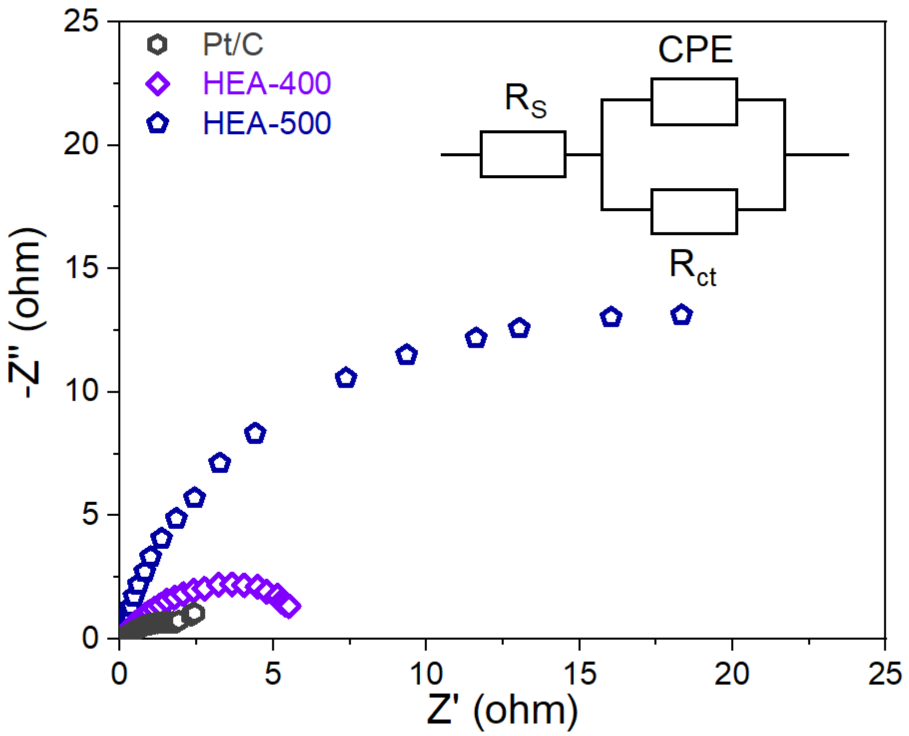

Supplementary Fig. 51. Nyquist plots of activated HEA-400 (HEA-400-5000), activated HEA-500 (HEA-500-2000), and Pt/C, the inset shows the equivalent electrical circuit diagram, where R_s, R_{ct} and CPE denote the solution resistance, charge transfer resistance and constant phase element, respectively.

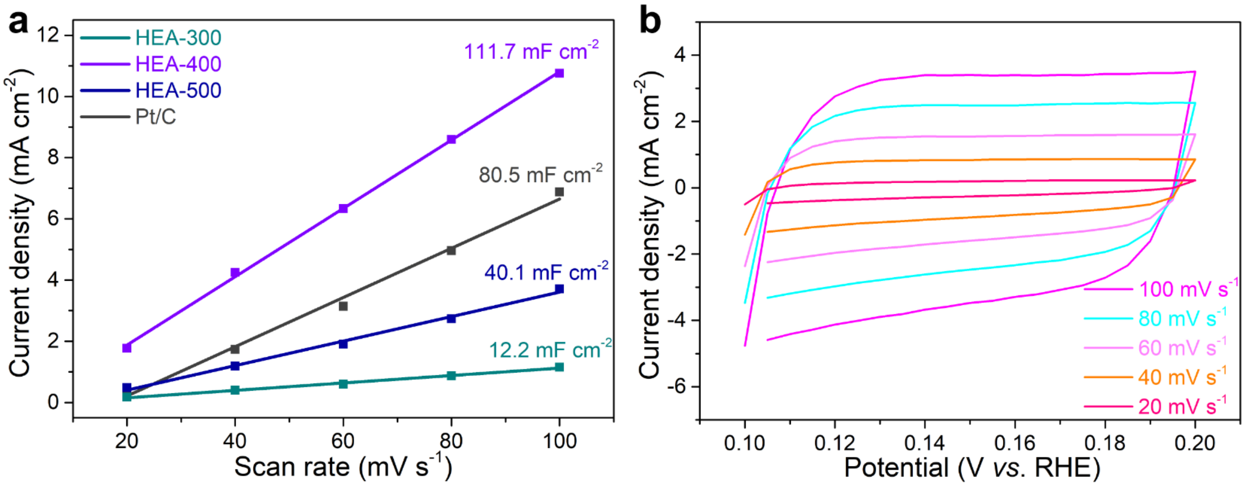

Supplementary Fig. 52. (a) Plots of capacitive currents with various scan rates for activated HEA-400 (HEA-400-5000), activated HEA-500 (HEA-500-2000), and Pt/C. (b) Detailed CV data for Pt/C to determine the double layer capacitance.

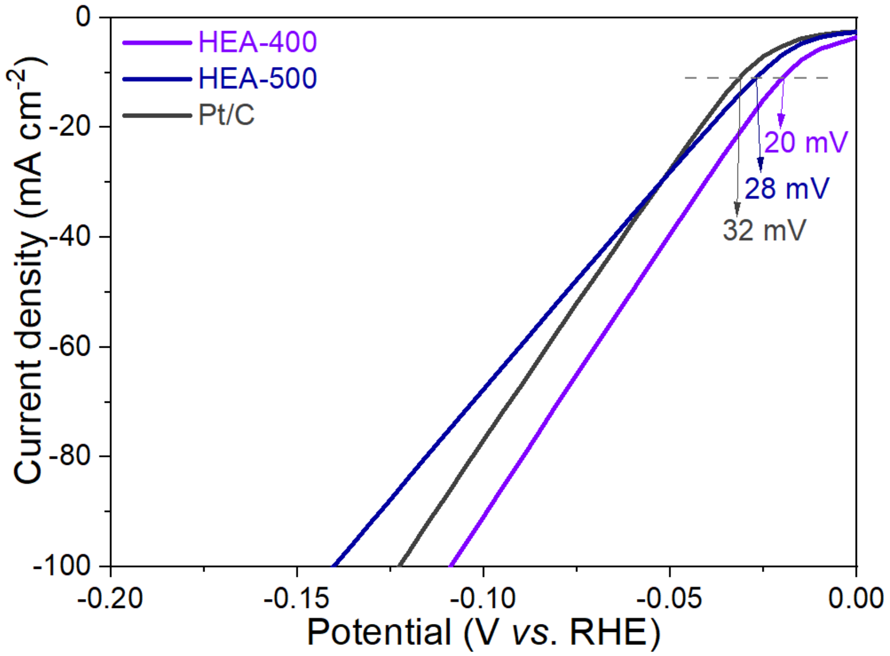

Supplementary Fig. 53. Polarization curves of activated HEA-400 (HEA-400-5000), activated HEA-500 (HEA-500-2000), and Pt/C without *iR*-compensation.

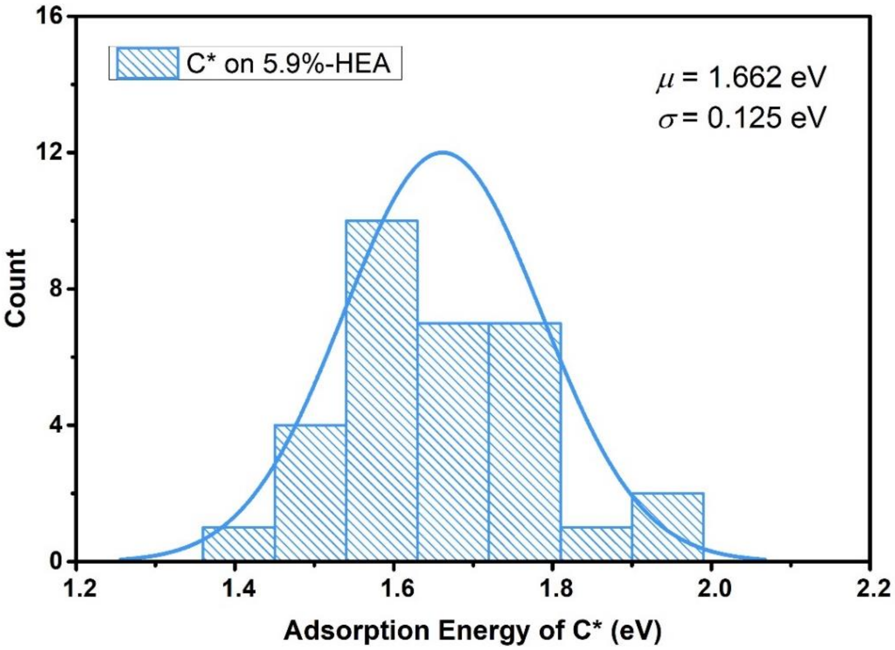

Supplementary Fig. 54. Gaussian distribution of adsorption energy of C* on 5.9%-HEA.

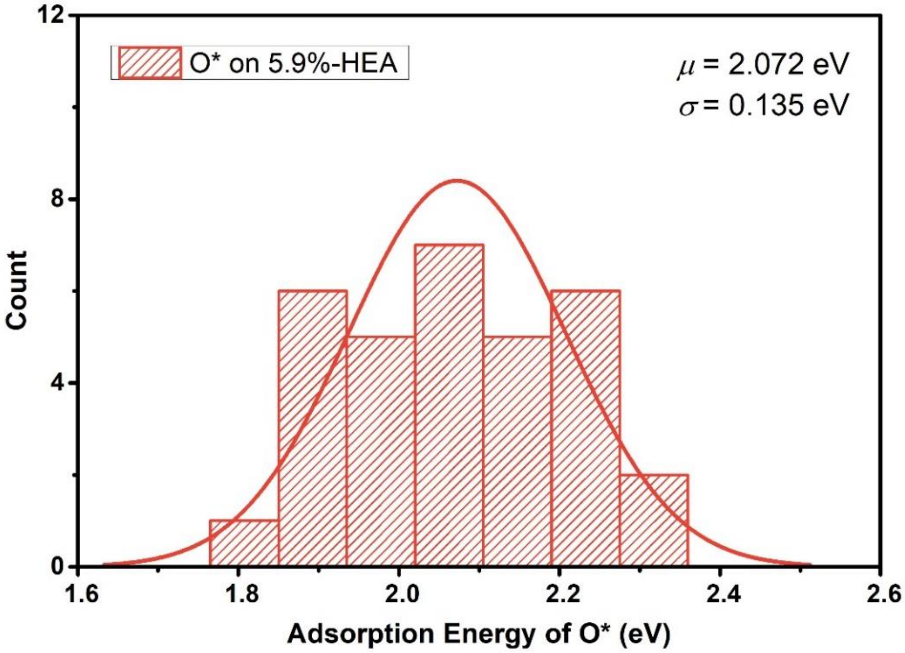

Supplementary Fig. 55. Gaussian distribution of adsorption energy of O* on 5.9%-HEA.

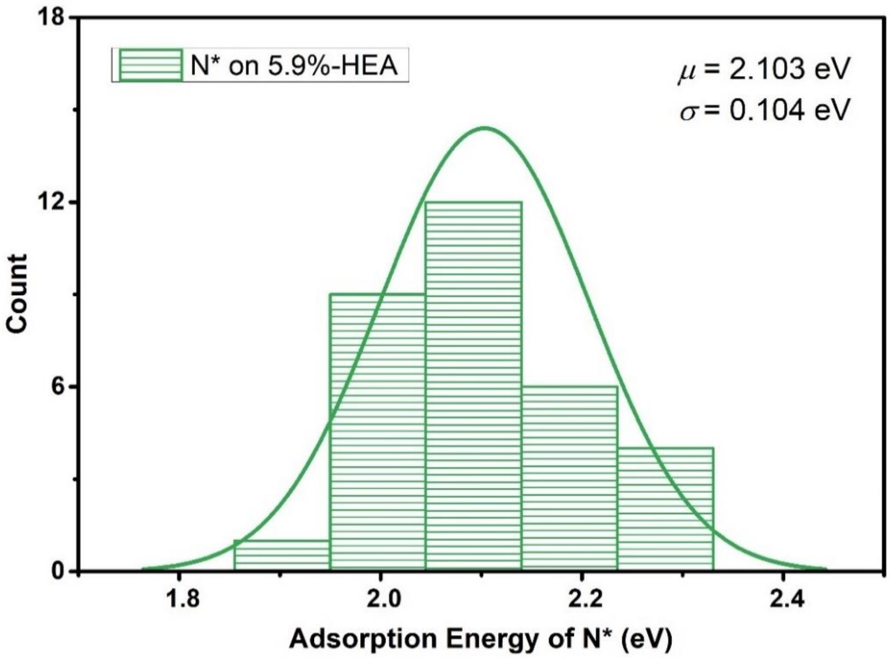

Supplementary Fig. 56. Gaussian distribution of adsorption energy of N* on 5.9%-HEA.

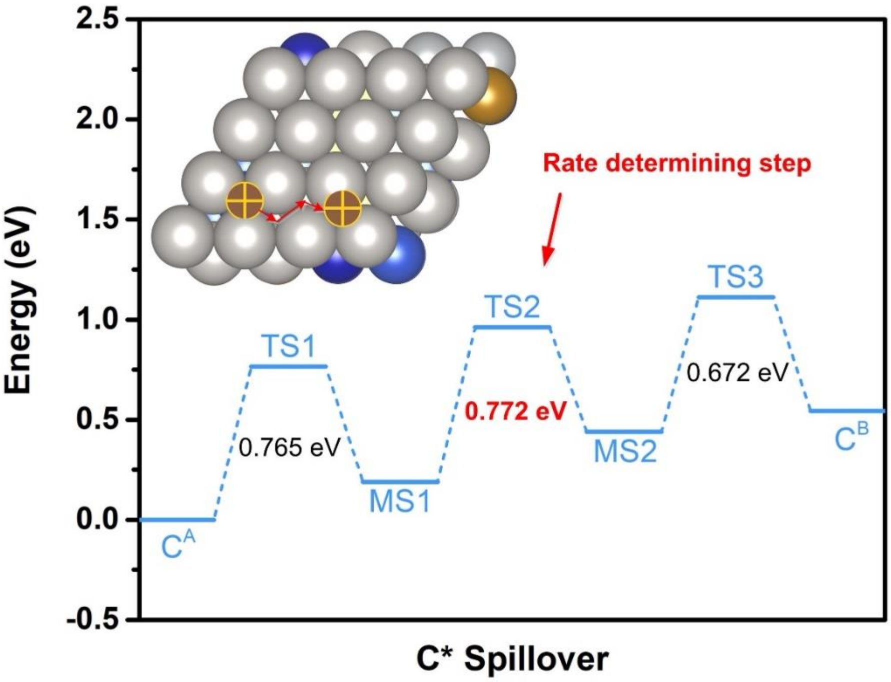

Supplementary Fig. 57. C* spillover on 5.9%-HEA (111).

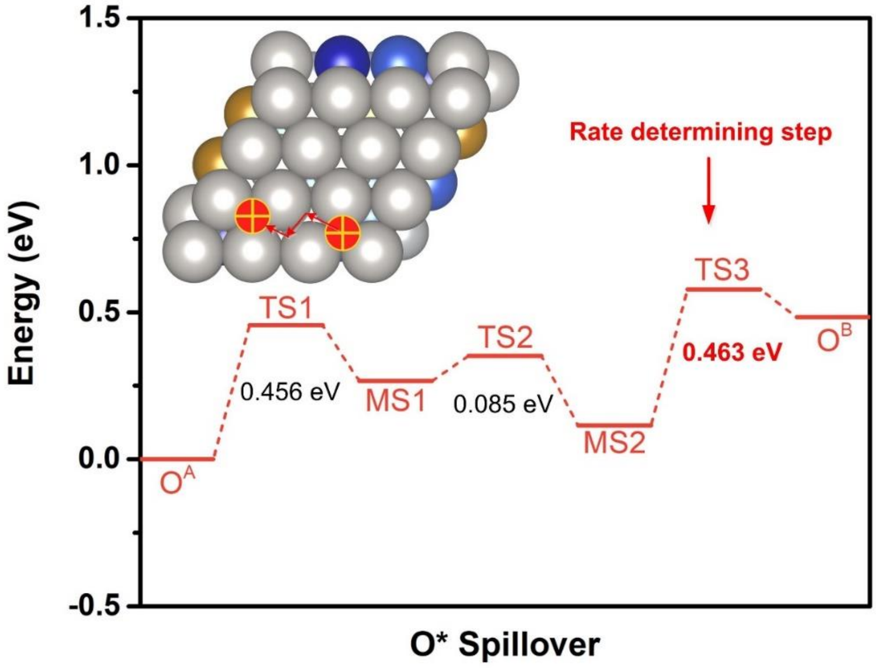

Supplementary Fig. 58. O* spillover on 5.9%-HEA (111).

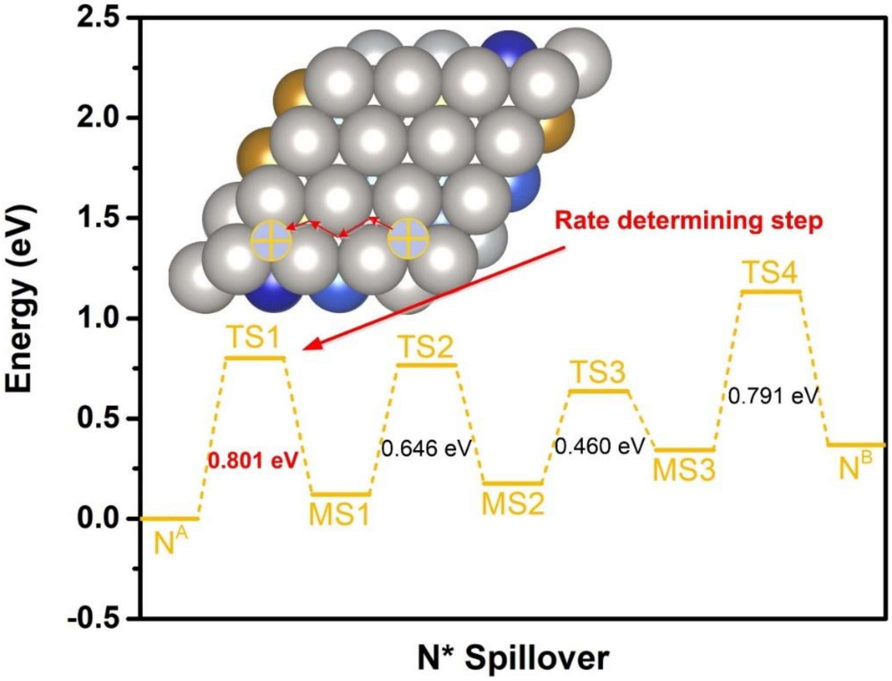

Supplementary Fig. 59. N* spillover on 5.9%-HEA (111).

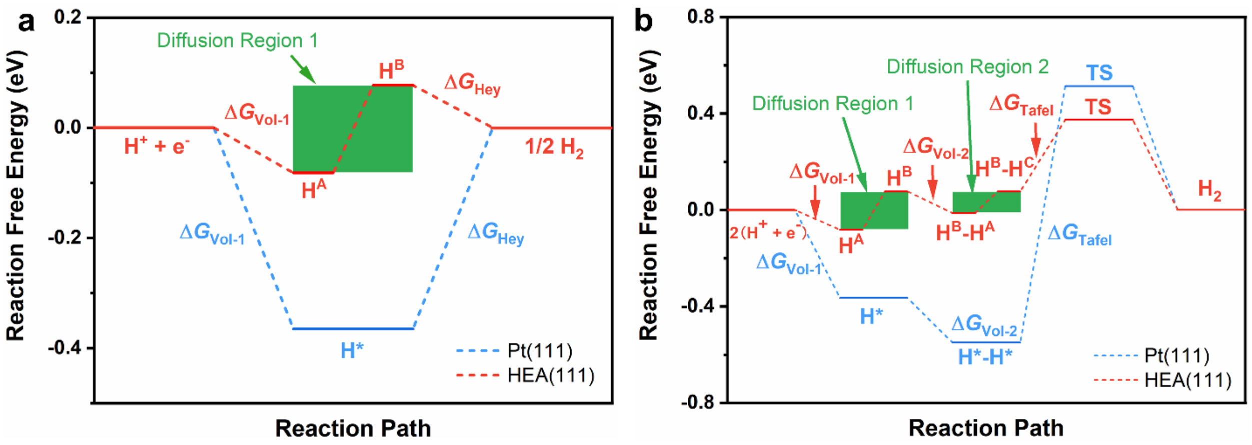

Supplementary Fig. 60. (a)Volmer-Heyrovsky mechanism and (b) Volmer-Tafel mechanism of HER on 5.9%-HEA (111) and Pt (111) under the high accuracy calculation parameters (k-point: 4 × 4 × 1; energy convergence: 1 × 10^{-8} eV; force convergence: 0.01 eV/Å).

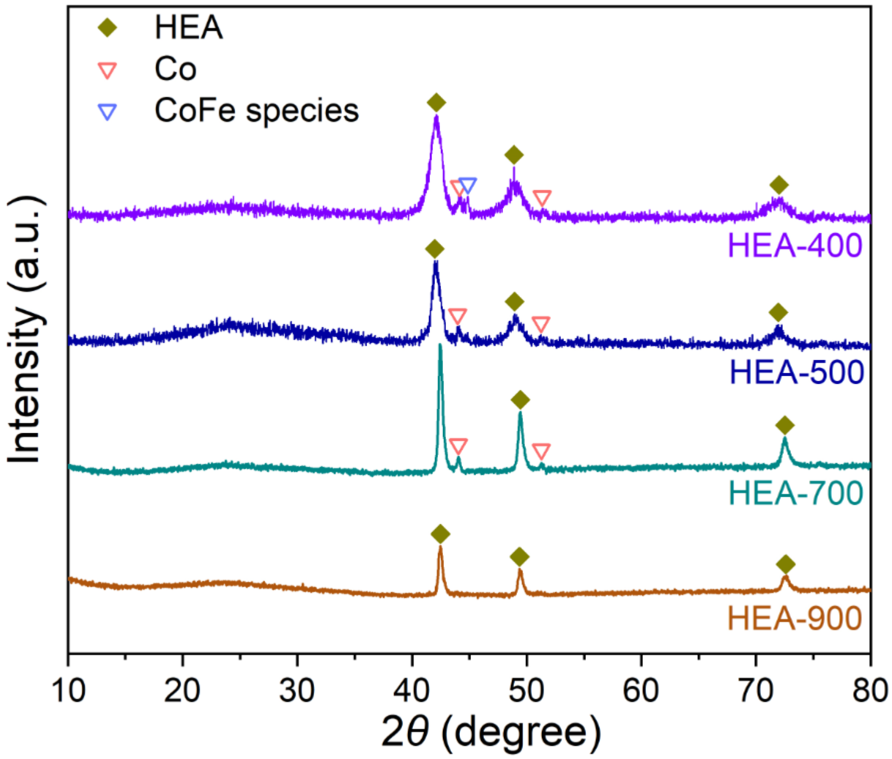

Supplementary Fig. 61. XRD patterns of HEA-400, HEA-500, HEA-700 and HEA-900.

Supplementary Note 10: All the samples present 3 main peaks corresponding to the (111), (200) and (220) planes of a face-centered cubic HEA structure. For HEA-400, three impure phase peaks are detected. The peaks at 44.2° and 51.5° correspond to the (111) and (200) planes of Co (JCPDS 89-7093), the peak at 44.8° corresponds to the (110) plane of CoFe species (maybe CoFe, JCPDS 44-1433 or Co_3Fe_7 JCPDS 48-1816). When increasing the annealing temperature, CoFe phases disappear while Co peaks still exist in HEA-500 and HEA-700. A well-defined HEA is synthesized when further increasing the annealing temperature to 900 °C. The detected (111) planes for HEA-400, HEA-500, HEA-700 and HEA-900 locate at 42.1°, 42.1°, 42.4° and 42.5°, respectively. Positive shifts of the (111) peaks as the annealing temperature increases imply the greater compressive strain in the HEAs. According to DFT results, the HEA with a strain of 6.8% should show a higher catalytic performance for HER. Besides, the HEA particle size also increases with the annealing temperature, a large particle size is not conducive to an excellent HER activity. Therefore, although the HEA-900 presents a well-defined HEA-structure, the excessive particle size (70 ~ 250 nm, see Supplementary Fig. 62a) and compressive strain (9.7%, based on the standard lattice spacing of 0.226 nm for Pt (111), see Supplementary Fig. 62b) make HEA-900 not the best choice for HER.

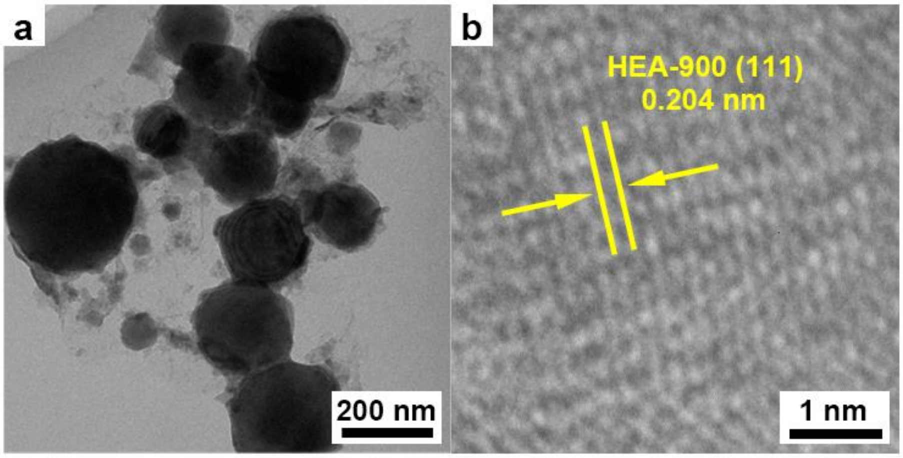

Supplementary Fig. 62. (a) TEM image of HEA-900. (b) HRTEM image of HEA-900.

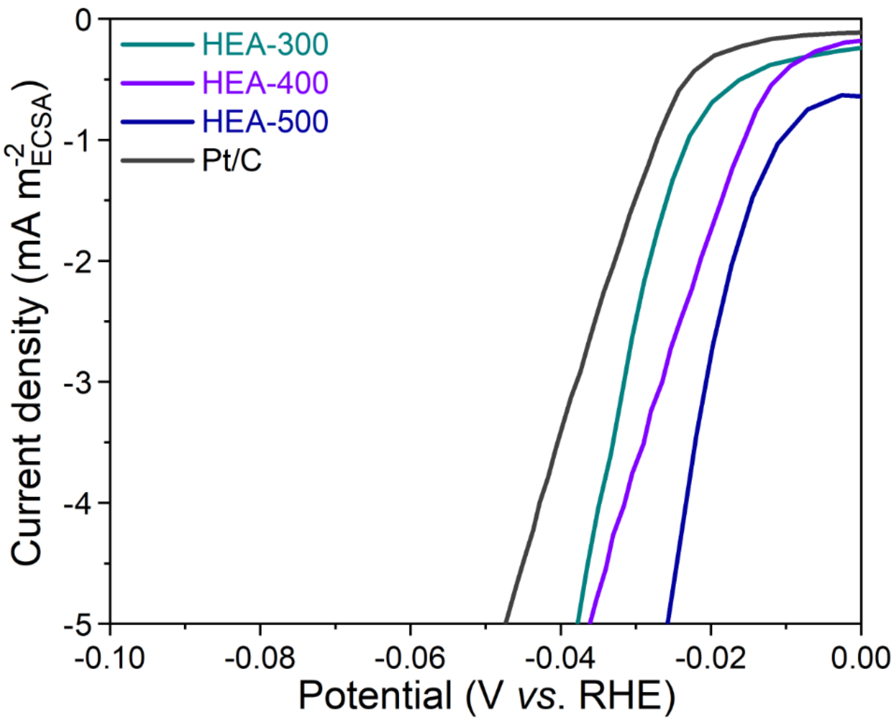

Supplementary Fig. 63. Polarization curves normalized to ECSA for HEA-300, HEA-400, HEA-500 and Pt/C.

Supplementary Table 1. Reaction free energy values of all steps during HER on 5.9%-HEA (111), HEA (111), 5.9%-Pt (111) and Pt (111).

Reactions	5.9%-HEA (111)	HEA (111)	5.9%-Pt (111)	Pt (111)
$\Delta G_{Vol\text{-}1}$ (eV)	-0.099 (-0.109[a])	-0.358	-0.270	-0.375
$\Delta G_{Vol\text{-}2}$ (eV)	-0.091 (-0.087[a])	-0.297	-0.114	-0.201
ΔG_{Hey} (eV)	-0.075 (-0.039[a])	0.197	0.270	0.375
ΔG_{Tafel} (eV)	0.297 (0.370[a])	0.578	1.006	1.128

The symbol of [a] indicates the reaction process of HER on another randomly active center of 5.9%-HEA (111) (see Supplementary Fig. 10).

Supplementary Table 2. Contents of Pt, Fe, Co, Ni and Cu in the electrolytes after different cycles of CV activation for HEA-400.

**Electrolytes**	**Pt (mg/L)**	**Fe (mg/L)**	**Co (mg/L)**	**Ni (mg/L)**	**Cu (mg/L)**
HEA after 100 CV cycles	0	0.0587	0.1299	0.0635	0.0255
HEA after 2000 CV cycles	0	0.1040	0.1480	0.0928	0.1546
HEA after 5000 CV cycles	0	0.1112	0.1514	0.0951	0.1656
HEA after 10000 CV cycles	0	0.1009	0.1560	0.1048	0.1722

Supplementary Table 3. Comparison of HER performance among some reported catalysts in 0.5 M H_2SO_4.

Catalysts	Overpotential at 10 mA cm^{-2} (mV)	Tafel slope (mV dec^{-1})	Reference
**PtFeCoNiCu HEA**	**10.8**	**28.1**	**This work**
Pt/MoS_2	14	96	(*9*)
PtSA/NT/NF	24	30	(*10*)
ALDPt/NGNs	48	29	(*11*)
Pt/MoS_2	32.9	25	(*12*)
$AL-Pt/Pd_3Pb$	13.8	18	(*13*)
Pt/AG	12	29.3	(*14*)
$Mo_2TiC_2T_x-Pt_{SA}$	30	30	(*15*)
Pt_1/OLC	38	36	(*16*)
$Pt/np-Co_{0.85}Se$	55	35	(*17*)
Rh/MoS_2	67	54	(*18*)
Pt/PCM	105	65.3	(*19*)
RuCoP	11	31	(*20*)
Ir/CON	13.6	32	(*21*)
$Pt/RuCeO_x$	41	31	(*22*)
Pt/WO_3	39	32.9	(*23*)
Ir/SiNW	22	20	(*24*)
EG-Pt/CoP	21	42.5	(*25*)
PbPtCuNiP	62	44.6	(*26*)
K_2PtCl_4/NC	11	21	(*27*)
Pt/MC	27.3	26	(*28*)

Supplementary Table 4. Reaction free energy values of all steps during HER on 5.9%-HEA (111) and Pt (111) with low (kpoint: 2 × 2 × 1; energy convergence: 1 × 10^{-5} eV; force convergence: 0.05 eV/Å)/high (kpoint: 4 × 4 × 1; energy convergence: 1 × 10^{-8} eV; force convergence: 0.01 eV/Å) precision calculation parameters.

Reactions	Catalysts	Low Accuracy	High Accuracy
$\Delta G_{\text{Vol-1}}$ (eV)	Pt (111)	-0.375	-0.365
	5.9%-HEA (111)	-0.099	-0.082
$\Delta G_{\text{Vol-2}}$ (eV)	Pt (111)	-0.201	-0.185
	5.9%-HEA (111)	-0.091	-0.089
ΔG_{Hey} (eV)	Pt (111)	0.375	0.365
	5.9%-HEA (111)	-0.075	-0.077
ΔG_{Tafel} (eV)	Pt (111)	1.128	1.063
	5.9%-HEA (111)	0.297	0.297

Supplementary References

1. S. Wang, W. Xu, Y. Zhu, Q. Luo, C. Zhang, S. Tang, Y. Du, Synthesis of structurally stable and highly active $PtCo_3$ ordered nanoparticles through an easily operated strategy for enhanced oxygen reduction reaction. *ACS Appl. Mater. Interfaces* **13**, 827-835 (2021).
2. J. D. Benck, Z. B. Chen, L. Y. Kuritzky, A. J. Forman, T. F. Jaramillo, Amorphous molybdenum sulfide catalysts for electrochemical hydrogen production: insights into the origin of their catalytic activity. *ACS Catal.* **2**, 1916-1923 (2012).
3. C. C. L. McCrory, S. Jung, I. M. Ferrer, S. M. Chatman, J. C. Peters, T. F. Jaramillo, Benchmarking hydrogen evolving reaction and oxygen evolving reaction electrocatalysts for solar water splitting devices. *J. Am. Chem. Soc.* **137**, 4347-4357 (2015).
4. G. Kresse, J. Furthmuller, Efficient iterative schemes for ab initio total-energy calculations using a plane-wave basis set. *Phys. Rev. B* **54**, 11169 (1996).
5. P. E. Blochl, Projector augmented-wave method. *Phys. Rev. B* **50**, 17953-17979 (1994).
6. J. P. Perdew, K. Burke, M. Ernzerhof, Generalized gradient approximation made simple. *Phys. Rev. Lett.* **77**, 3865 (1996).
7. S. Grimme, J. Antony, S. Ehrlich, H. Krieg, A consistent and accurate ab initio parametrization of density functional dispersion correction (DFT-D) for the 94 elements H-Pu. *J. Chem. Phys.* **132**, 154104 (2010).
8. G. Henkelman, B. P. Uberuaga, H. Jonsson, A climbing image nudged elastic band method for finding saddle points and minimum energy paths. *J. Chem. Phys.* **113**, 9901-9904 (2000).
9. J. Deng, H. B. Li, J. P. Xiao, Y. C. Tu, D. H. Deng, H. X. Yang, H. F. Tian, J. Q. Li, P. J. Ren, X. H. Bao, Triggering the electrocatalytic hydrogen evolution activity of the inert two-dimensional MoS_2 surface via single-atom metal doping. *Energy Environ. Sci.* **8**, 1594-1601 (2015).
10. L. H. Zhang, L. L. Han, H. X. Liu, X. J. Liu, J. Luo, Potential-cycling synthesis of single Pt atoms for efficient hydrogen evolution in neutral media. *Angew. Chem. Int. Edit.* **56**, 13694-13698 (2017).
11. N. C. Cheng, S. Stambula, D. Wang, M. N. Banis, J. Liu, A. Riese, B. W. Xiao, R. Y. Li, T. K. Sham, L. M. Liu, G. A. Botton, X. L. Sun, Platinum single-atom and cluster catalysis of the hydrogen evolution reaction. *Nat. Commun.* **7**, 13638 (2016).
12. Z. X. Chen, K. Leng, X. X. Zhao, S. Malkhandi, W. Tang, B. B. Tian, L. Dong, L. R. Zheng, M. Lin, B. S. Yeo, K. P. Loh, Interface confined hydrogen evolution reaction in zero valent metal nanoparticles-intercalated molybdenum disulfide. *Nat. Commun.* **8**, 14548 (2017).
13. Y. C. Yao, X. K. Gu, D. S. He, Z. J. Li, W. Liu, Q. Xu, T. Yao, Y. Lin, H. J. Wang, C. M. Zhao, X. Q. Wang, P. Q. Yin, H. Li, X. Hong, S. Q. Wei, W. X. Li, Y. D. Li, Y. Wu, Engineering the electronic structure of submonolayer Pt on intermetallic Pd_3Pb via charge transfer boosts the hydrogen evolution reaction. *J. Am. Chem. Soc.* **141**, 19964-19968 (2019).
14. S. H. Ye, F. Y. Luo, Q. L. Zhang, P. Y. Zhang, T. T. Xu, D. S. He, L. C. Guo, Y. Zhang, C. X. He, X. P. Ouyang, Q. Wang, M. Gu, J. H. Liu, X. L. Sun, Highly stable single Pt atomic sites anchored on aniline-stacked graphene for hydrogen evolution reaction. *Energy Environ. Sci.* **12**, 1000-1007 (2019).
15. J. Q. Zhang, Y. F. Zhao, X. Guo, C. Chen, C. L. Dong, R. S. Liu, C. P. Han, Y. D. Li, Y. Gogotsi, G. X. Wang, Single platinum atoms immobilized on an MXene as an efficient catalyst for the hydrogen evolution reaction. *Nat. Catal.* **1**, 985-992 (2018).
16. D. B. Liu, X. Y. Li, S. M. Chen, H. Yan, C. D. Wang, C. Q. Wu, Y. A. Haleem, S. Duan, J. L. Lu, B. H. Ge, P. M. Ajayan, Y. Luo, J. Jiang, L. Song, Atomically dispersed platinum

supported on curved carbon supports for efficient electrocatalytic hydrogen evolution. *Nat. Energy* **4**, 512-518 (2019).

17. K. Jiang, B. Y. Liu, M. Luo, S. C. Ning, M. Peng, Y. Zhao, Y. R. Lu, T. S. Chan, F. M. F. Groot, Y. W. Tan, Single platinum atoms embedded in nanoporous cobalt selenide as electrocatalyst for accelerating hydrogen evolution reaction. *Nat. Commun.* **10**, 1743 (2019).
18. X. Y. Meng, C. Ma, L. Z. Jiang, R. Si, X. G. Meng, Y. C. Tu, L. Yu, X. H. Bao, D. H. Deng, Distance synergy of MoS_2-confined Rhodium atoms for highly efficient hydrogen evolution. *Angew. Chem. Int. Edit.* **59**, 10502-10507 (2020).
19. H. B. Zhang, P. F. An, W. Zhou, B. Y. Guan, P. Zhang, J. C. Dong, X. W. Lou, Dynamic traction of lattice-confined platinum atoms into mesoporous carbon matrix for hydrogen evolution reaction. *Sci. Adv.* **4**, eaao6657 (2018).
20. J. Y. Xu, T. F. Liu, J. J. Li, Y. F. Liu, B. S. Zhang, D. H. Xiong, I. Amorim, W. Li, L. F. Liu, Boosting the hydrogen evolution performance of ruthenium clusters through synergistic coupling with cobalt phosphide. *Energy Environ. Sci.* **11**, 1819-1827 (2018).
21. J. Mahmood, M. A. R. Anjum, S. H. Shin, I. Ahmad, H. J. Noh, S. J. Kim, H. J. Jeong, J. S. Lee, J. B. Baek, Encapsulating iridium nanoparticles inside a 3D cage-like organic network as an efficient and durable catalyst for the hydrogen evolution reaction. *Adv. Mater.* **30**, 1805606 (2018).
22. T. T. Liu, W. B. Gao, Q. Q. Wang, M. L. Dou, Z. P. Zhang, F. Wang, Selective loading of atomic platinum on a $RuCeO_x$ support enables stable hydrogen evolution at high current densities. *Angew. Chem. Int. Edit.* **59**, 20423-20427 (2020).
23. C. Xie, W. Chen, S. Q. Du, D. F. Yan, Y. Q. Zhang, J. Chen, B. Liu, S. Y. Wang, In-situ phase transition of WO_3 boosting electron and hydrogen transfer for enhancing hydrogen evolution on Pt. *Nano Energy* **71**, 104653 (2020).
24. M. Q. Sheng, B. B. Jiang, B. Wu, F. Liao, X. Fan, H. P. Lin, Y. Y. Li, Y. Lifshitz, S. T. Lee, M. W. Shao, Approaching the volcano top: Iridium/Silicon nanocomposites as efficient electrocatalysts for the hydrogen evolution reaction. *ACS Nano* **13**, 2786-2794 (2019).
25. J. Y. Li, H. X. Liu, W. Y. Gou, M. K. Zhang, Z. M. Xia, Z. Zhang, C. R. Chang, Y. Y. Ma, Y. Q. Qu, Ethylene-glycol ligand environment facilitates highly efficient hydrogen evolution of Pt/CoP through proton concentration and hydrogen spillover. *Energy Environ. Sci.* **12**, 2298-2304 (2019).
26. Z. Jia, K. Nomoto, Q. Wang, C. Kong, L. G. Sun, L. C. Zhang, S. X. Liang, J. Lu, J. J. Kruzic, A self-supported high-entropy metallic glass with a nanosponge architecture for efficient hydrogen evolution under alkaline and acidic conditions. *Adv. Funct. Mater.* **31**, 2101586 (2021).
27. H. Y. Jin, S. Sultan, M. R. Ha, J. N. Tiwari, M. G. Kim, K. S. Kim, Simple and scalable mechanochemical synthesis of noble metal catalysts with single atoms toward highly efficient hydrogen evolution. *Adv. Funct. Mater.* **30**, 2000531 (2020).
28. H. H. Wei, K. Huang, D. Wang, R. Y. Zhang, B. H. Ge, J. Y. Ma, B. Wen, S. Zhang, Q. Y. Li, M. Lei, C. Zhang, J. Irawan, L. M. Liu, H. Wu, Iced photochemical reduction to synthesize atomically dispersed metals by suppressing nanocrystal growth. *Nat. Commun.* **8**, 1490 (2017).
